# Culturable diversity of bacterial endophytes associated with medicinal plants of the Western Ghats, India

**DOI:** 10.1093/femsec/fiaa147

**Published:** 2020-07-25

**Authors:** Gordon Webster, Alex J Mullins, Edward Cunningham-Oakes, Arun Renganathan, Jamuna Bai Aswathanarayan, Eshwar Mahenthiralingam, Ravishankar Rai Vittal

**Affiliations:** Microbiomes, Microbes and Informatics Group, Organisms and Environment Division, School of Biosciences, Cardiff University, Cardiff, CF10 3AX, Wales, UK; Microbiomes, Microbes and Informatics Group, Organisms and Environment Division, School of Biosciences, Cardiff University, Cardiff, CF10 3AX, Wales, UK; Microbiomes, Microbes and Informatics Group, Organisms and Environment Division, School of Biosciences, Cardiff University, Cardiff, CF10 3AX, Wales, UK; Department of Studies in Microbiology, University of Mysore, Karnataka, 570006, Mysore, India; Department of Studies in Microbiology, University of Mysore, Karnataka, 570006, Mysore, India; Microbiomes, Microbes and Informatics Group, Organisms and Environment Division, School of Biosciences, Cardiff University, Cardiff, CF10 3AX, Wales, UK; Department of Studies in Microbiology, University of Mysore, Karnataka, 570006, Mysore, India

**Keywords:** endophytic bacteria, antimicrobials, *Bacillus*, medicinal plants, bacterial genomes, biosynthetic gene clusters

## Abstract

Bacterial endophytes are found in the internal tissues of plants and have intimate associations with their host. However, little is known about the diversity of medicinal plant endophytes (ME) or their capability to produce specialised metabolites that may contribute to therapeutic properties. We isolated 75 bacterial ME from 24 plant species of the Western Ghats, India. Molecular identification by 16S rRNA gene sequencing grouped MEs into 13 bacterial genera, with members of Gammaproteobacteria and Firmicutes being the most abundant. To improve taxonomic identification, 26 selected MEs were genome sequenced and average nucleotide identity (ANI) used to identify them to the species-level. This identified multiple species in the most common genus as *Bacillus*. Similarly, identity of the Enterobacterales was also distinguished within *Enterobacter* and *Serratia* by ANI and core-gene analysis. AntiSMASH identified non-ribosomal peptide synthase, lantipeptide and bacteriocin biosynthetic gene clusters (BGC) as the most common BGCs found in the ME genomes. A total of five of the ME isolates belonging to *Bacillus*, *Serratia* and *Enterobacter* showed antimicrobial activity against the plant pathogen *Pectobacterium carotovorum*. Using molecular and genomic approaches we have characterised a unique collection of endophytic bacteria from medicinal plants. Their genomes encode multiple specialised metabolite gene clusters and the collection can now be screened for novel bioactive and medicinal metabolites.

## Teaser

A collection of bacterial endophytes isolated from a number of medicinal plants of the Western Ghats, India were investigated for their capability to produce specialised metabolites that may contribute to therapeutic properties.

## INTRODUCTION

Multiple countries use indigenous plants as traditional remedies for treatment of injury or disease. In the Indian traditional medicinal system of Ayurveda and other similar practices, leaves, roots, seeds and fruits are commonly used as alternative medicines. *Garcinia indica* (Baliga *et al*. 2011), *Salacia chinensis* (Deokate and Khadabadi 2012) and *Alstonia scholaris* (Ganjewala and Gupta 2013) are examples of Indian medicinal plant species described to have multiple therapeutic properties. *Garcinia indica*, commonly known as the kokum tree, produces fruits which are used in Ayurvedic medicine for its antimicrobial, antiulcer, anticancer and antiobesity properties, as well as being able to ease inflammatory and pain-related issues (Baliga *et al*. 2011). The roots of the *Salacia chinensis* herb tree have also been exploited for beneficial properties in treating tooth decay, ulcers, obesity and skin conditions (Deokate and Khadabadi 2012). Multiple parts of the Indian devil tree, *Alstonia scholaris*, such as leaves, follicles and latex show extensive antimicrobial and antioxidant properties (Ganjewala and Gupta 2013). Recently, the medicinal plant-associated microbiome, and especially the interaction between the complex community of endophytic microorganisms (endomicrobiome; Köberl *et al*. 2013) have been attributed to these antimicrobial (Martinez-Klimova, Rodríguez-Peña and Sánchez 2017) and bioactive properties through the metabolites they produce (Gouda *et al*. 2016; Ek-Ramos *et al*. 2019). Endophytic bacteria isolated from traditional Chinese medicinal plants used as anticancer therapy were screened for bioactivity and all isolates exhibited either cytotoxic, antibacterial or antifungal activities in at least one assay (Miller *et al*. 2012b).

Endophytic microorganisms (endophytes) are bacteria or fungi that colonise the intercellular and/or intracellular spaces of plants, often living in a symbiotic relationship (Hardoim *et al*. 2015). Endophytes are known to promote plant growth and nutrient gain, improve yield and aid the plant to survive in harsh conditions when under stress or attack from pathogens (Ryan *et al*. 2008; Hardoim *et al*. 2015; Santoyo *et al*. 2016). It is thought for this reason many endophytes produce a range of unique specialised metabolites, such as peptides, polyketides and alkaloids, to aid the plants immune response and prevent colonisation by pathogens and other microbes. Natural products from endophytes frequently possess bioactivities such as antimicrobial, antifungal, anticarcinogen, immunosuppressant and antioxidant (Zhang, Song and Tan 2006; Akinsanya *et al*. 2015; Sharma *et al*. 2020) and their investigation offers huge potential in identifying new pharmaceutical compounds.

However, whilst nearly all plants are thought to contain endophytes, very little is known about the diversity of endophytes in traditional Indian medicinal plant species. India is considered to be one of the 16 mega diversity countries in the world with around 17 500 higher plants species, of which 4050 plants are found in the Western Ghats (Pascal, Ramesh and De Franceschi 2004), many of which are used in the treatment of infection, disease, wounds and injuries (Ayyanar and Ignacimuthu^2011^). In this study we aimed to determine the culturable diversity of bacterial endophytes present within a large collection of plant species taken from the Western Ghats region. Plants were chosen based on their ethnobotanical usage, being endemic to the region or were found growing within biodiversity rich areas (Strobel and Daisy 2003). Endophytes were then isolated from the leaves (the main plant part of medicinal value) from 24 plant species, initially identified by 16S rRNA gene analysis, and then followed up with whole genome sequencing for finer resolution of their taxonomy. Selected endophytes were also investigated further for their specialised metabolite potential via a genome mining and antimicrobial bioactivity analysis.

## MATERIALS AND METHODS

### Sample site and plant material collection

The Western Ghats (or Sahyadri) is a mountain region that covers an area of around 140 000 km^2^ and 1600 km in length running parallel to the western coast of the Indian peninsula from the river Tapti in the North to Kanyakumari in the South (Reddy, Jha and Dadhwal 2016). It traverses parts of six states, Kerala, Tamil Nadu, Karnataka, Goa, Maharashtra and Gujarat and is a UNESCO World Heritage Site and one of the eight "hottest hotspots" of biological diversity in the world (Myers *et al*. 2000). It has non-equatorial tropical evergreen forests which hosts at least 325 globally threatened species of flora and fauna (UNESCO). The database on ethnomedicinal plants of Western Ghats lists 500 plants from 115 families that have been used to prepare around 600 different medicinal formulations as listed by the Indian Council of Medical Research (Project by SD Kholkute, 2005–2008, submitted to ICMR). However, it is estimated that the true number of medicinal plants in the Western Ghats is >700 species with many being endemic and listed as endangered in the International Union for Conservation of Nature (IUCN) Red List of threatened species (https://www.iucnredlist.org/).

Leaf samples (and one fruit sample) from 24 plant species (covering 19 plant genera; Figure S1a and Table S1, Supporting Information) were collected from two sites of the Western Ghats and one site of Mysore in Karnataka, India between 5th July and 28th August 2017. All samples were then transported to the laboratory in sterile polypropylene bags and processed within 24 h of collection. Each plant was identified by referring to literature, herbarium specimens, consulting with taxonomists and searching databases including The Western Ghats (India Biodiversity Portal), Sahyadri (Western Ghats Biodiversity Information System) and Digital Flora of Karnataka. Samples of the plant species were preserved in the Herbarium of Department of Studies in Microbiology, University of Mysore, India.

### Isolation of endophytic bacteria

Samples were washed with distilled water and surface sterilised using the following procedure: 0.1% (w/v) HgCl_2_ solution for 1 min, sterile water for 1 min, 90% (v/v) ethanol for 2 min and finally washed again with sterile water. Leaf samples were then cut into segments of approximately 0.5 cm^2^ using a sterile scalpel and placed onto Luria-Bertani (LB) agar (HiMedia Laboratories, Mumbai, India) plates and incubated at ambient temperature in the dark. To ensure bacterial growth was only obtained from plant endophytes, one additional LB agar plate for each plant was also incubated with uncut surface sterilised leaves as a control (Martinez-Klimova, Rodríguez-Peña and Sánchez 2017). No growth from plant epiphytic bacteria was observed. During incubation, inoculated plates were frequently observed for bacterial growth at the cut-ends of the leaf tissue and emerging bacteria were transferred onto fresh LB agar. Bacterial endophytes were streaked, and individual colonies were selected and sub-cultured three times to obtain pure bacterial cultures on LB agar. Each bacterial isolate was transferred separately to LB agar slopes and stored at 4°C for further study. The cultures were maintained at the University of Mysore for characterisation and elucidation of bioactive compounds, while phylogenetic analysis and genome sequencing of endophytes was performed at Cardiff University. All bacterial cultures isolated in this study are available from the laboratory collection held at the Department of Studies in Microbiology, University of Mysore, India by request from the corresponding author.

For molecular characterisation analysis, bacterial isolates were revived on TSA (Tryptone Soy agar; Oxoid, Basingstoke, UK) plates at 30°C, sub-cultured three times and checked for purity, except isolates ME7 and ME8 which grew better on Reasoner's 2A agar (R2A agar, Oxoid). Pure cultures were stored at −80°C in 8% (v/v) dimethyl sulfoxide (DMSO) and tryptone soya broth (TSB) or R2A.

### 16S rRNA gene diversity and phylogenetic analysis of bacterial endophytes

DNA was extracted from 10 µL of an overnight culture (grown in TSB or R2A at 30°C) with 100 µL of 5% (w/v) Chelex 100 resin (Walsh, Metzger and Higuchi 1991) by undergoing two cycles of boiling and freezing (5 mins each) as described (Parkes *et al*. 2010). The crude DNA extract was then used as template in a 16S rRNA gene PCR with bacterial primers 27F and 907R (Webster *et al*. 2006). All 16S rRNA gene PCR amplicons were analysed by 1.2% (w/v) agarose gel electrophoresis, purified and sequenced at Eurofins Genomics (https://www.eurofinsgenomics.eu/en/home/) by Sanger sequencing with primer 27F. Sequence chromatograms were analysed using Chromas version 2.6.6 (http://technelysium.com.au) and mixed sequences (suggestive that some isolates were not pure) were removed from further analysis resulting in 75 pure endophytic bacterial isolates (see Table 1).

**Table 1.** List of bacterial endophytes isolated from leaves of medicinal plant species sampled at different locations of the Western Ghats, Karnataka, India.

Endophytic bacterium^a^	Plant species isolated from	Sampling location	Identification by 16S rRNA gene similarity	Identification by average nucleotide identity (ANI)	Identification by Type strain Genome Server (TYGS)
ME1	*Memecylon malabaricum*	Bisle Ghat region	*Serratia sp*.		
ME3	*Memecylon malabaricum*	Bisle Ghat region	*Serratia sp*.		
ME4	*Aphanamixis polystachya*	Bisle Ghat region	*Bacillus sp*.		
**ME5**	***Aphanamixis polystachya***	**Bisle Ghat region**	***Bacillus sp*.**	***Bacillus thuringiensis***	***Bacillus paranthracis***
ME6	*Terminalia bellirica*	Bisle Ghat region	*Bacillus sp*.		
**ME7**	***Terminalia bellirica***	**Bisle Ghat region**	***Aureimonas sp*.**	***Aureimonas sp*.**	***Aureimonas sp*.**
ME8	*Terminalia bellirica*	Bisle Ghat region	*Aureimonas sp*.		
ME9	*Terminalia bellirica*	Bisle Ghat region	*Bacillus sp*.		
ME10	*Ventilago* sp.	Bisle Ghat region	*Enterobacter sp*.		
ME11	*Ventilago* sp.	Bisle Ghat region	*Bacillus sp*.		
**ME12**	***Terminalia paniculata***	**Bisle Ghat region**	***Curtobacterium sp*.**	***Curtobacterium sp*.**	***Curtobacterium sp*.**
**ME13**	***Terminaliapaniculata***	**Bisle Ghat region**	***Enterobacter sp*.**	***Enterobacterbugandensis***	***Enterobacterbugandensis***
ME14	*Aristolochia tagala*	Bisle Ghat region	*Enterobacter sp*.		
ME15	*Aristolochia tagala*	Bisle Ghat region	*Klebsiella sp*.		
ME16	*Aristolochia tagala*	Bisle Ghat region	*Enterobacter sp*.		
ME17A	*Aristolochia tagala*	Bisle Ghat region	*Klebsiella sp*.		
ME18	*Aristolochia tagala*	Bisle Ghat region	*Enterobacter sp*.		
ME19	*Aristolochia tagala*	Bisle Ghat region	*Enterobacter sp*.		
ME20	*Aristolochia tagala*	Bisle Ghat region	*Bacillus sp*.		
ME21	*Aristolochia tagala*	Bisle Ghat region	*Klebsiella sp*.		
ME23	*Garcinia xanthochymus*	Bisle Ghat region	*Bacillus sp*.		
**ME25**	***Aphanamixis polystachya***	**Bisle Ghat region**	***Bacillus sp*.**	***Bacillus taxi***	***Bacillus taxi***
**ME26**	***Ventilago* sp**.	**Bisle Ghat region**	***Curtobacterium sp*.**	***Curtobacterium sp*.**	***Curtobacterium sp*.**
**ME27**	***Aphanamixis polystachya***	**Bisle Ghat region**	***Acinetobacter sp*.**	***Acinetobacter lactucae***	***Acinetobacter lactucae***
ME28	*Salacia macrosperma*	Bisle Ghat region	*Bacillus sp*.		
ME29	*Salacia macrosperma*	Bisle Ghat region	*Bacillus sp*.		
**ME30**	***Garcinia xanthochymus***	**Bisle Ghat region**	***Klebsiella sp*.**	***Klebsiella pneumoniae***	***Klebsiella pneumoniae***
ME31	*Garcinia xanthochymus*	Bisle Ghat region	*Enterobacter sp*.		
ME32	*Ventilago* sp.	Bisle Ghat region	*Bacillus sp*.		
ME33	*Ventilago* sp.	Bisle Ghat region	*Brevibacillus sp*.		
**ME34**	***Ventilago* sp**.	**Bisle Ghat region**	***Enterobacter sp*.**	***Enterobacter bugandensis***	***Enterobacter bugandensis***
**ME35**	***Terminalia bellirica***	**Bisle Ghat region**	***Bacillus sp*.**	***Bacillus licheniformis***	***Bacillus licheniformis***
ME36	*Terminalia paniculata*	Bisle Ghat region	*Bacillus sp*.		
ME38	*Ventilago* sp.	Bisle Ghat region	*Bacillus sp*.		
					
**ME39**	***Pterocarpus santalinus***	**Mysore**	***Bacillus sp*.**	***Bacillus aryabhattai***	***Bacillus aryabhattai***
**ME40**	***Garcinia indica***	**Mysore**	***Bacillus sp*.**	***Bacillus megaterium***	***Bacillus sp*.**
**ME42**	***Pterocarpus santalinus***	**Mysore**	***Bacillus sp*.**	***Bacillus aryabhattai***	***Bacillus aryabhattai***
					
**ME43**	***Coscinium fenestratum***	**Mangaluru**	***Serratia sp*.**	***Serratia marcescens***	***Serratia sp*.**
**ME44**	***Coscinium fenestratum***	**Mangaluru**	***Enterobacter sp*.**	***Enterobacter asburiae***	***Enterobacter asburiae***
ME45	*Coscinium fenestratum*	Mangaluru	*Enterobacter sp*.		
ME46	*Coscinium fenestratum*	Mangaluru	*Enterobacter sp*.		
**ME47**	***Coscinium fenestratum***	**Mangaluru**	***Serratia sp*.**	***Serratia marcescens***	***Serratia sp*.**
ME51	*Coscinium fenestratum*	Mangaluru	*Stenotrophomonas sp*.		
ME53	*Coix lacryma-jobi*	Mangaluru	*Stenotrophomonas sp*.		
**ME55**	***Coix lacryma-jobi***	**Mangaluru**	***Stenotrophomonas sp*.**	***Stenotrophomonas pavanii***	***Stenotrophomonas pavanii***
ME56	*Coix lacryma-jobi*	Mangaluru	*Paenibacillus sp*.		
ME57	*Coix lacryma-jobi*	Mangaluru	*Klebsiella sp*.		
ME60	*Coix lacryma-jobi*	Mangaluru	*Bacillus sp*.		
ME62	*Coix lacryma-jobi*	Mangaluru	*Enterobacter sp*.		
**ME63**	***Coix lacryma-jobi***	**Mangaluru**	***Pseudomonas sp*.**	***Pseudomonas sp*.**	***Pseudomonas sp*.**
ME64	*Coix lacryma-jobi*	Mangaluru	*Enterobacter sp*.		
ME66	*Coix lacryma-jobi*	Mangaluru	*Klebsiella sp*.		
ME67	*Coix lacryma-jobi*	Mangaluru	*Stenotrophomonas sp*.		
ME68	*Salacia chinensis*	Mangaluru	*Klebsiella sp*.		
ME70	*Salacia chinensis*	Mangaluru	*Klebsiella sp*.		
ME71	*Salacia chinensis*	Mangaluru	*Stenotrophomonas sp*.		
ME72	*Salacia chinensis*	Mangaluru	*Klebsiella sp*.		
**ME73**	***Salacia chinensis***	**Mangaluru**	***Klebsiella sp*.**	***Klebsiella variicola***	***Klebsiella variicola***
ME74	*Salacia chinensis*	Mangaluru	*Enterobacter sp*.		
**ME75**	***Calophyllum inophyllum***	**Mangaluru**	***Bacillus sp*.**	***Bacillus aryabhattai***	***Bacillus sp*.**
**ME76**	***Calophyllum inophyllum***	**Mangaluru**	***Bacillus sp*.**	***Bacillus aryabhattai***	***Bacillus sp*.**
**ME78**	***Madhuca insignis***	**Mangaluru**	***Bacillus sp*.**	***Bacillus thuringiensis***	***Bacillus cereus***
**ME79**	***Madhuca insignis***	**Mangaluru**	***Klebsiella sp*.**	***Klebsiella variicola***	***Klebsiella variicola***
**ME81**	***Garcinia morella***	**Mangaluru**	***Erwinia sp*.**	***Pantoea sp*.**	***Pantoea sp*.**
ME83	*Apama siliquosa*	Mangaluru	*Klebsiella sp*.		
ME84	*Apama siliquosa*	Mangaluru	*Stenotrophomonas sp*.		
**ME86**	***Desmodium pulchellum***	**Mangaluru**	***Klebsiella sp*.**	***Klebsiella variicola***	***Klebsiella variicola***
ME87	*Barringtonia acutangula*	Mangaluru	*Enterobacter sp*.		
ME89	*Barringtonia acutangula* fruit	Mangaluru	*Enterobacter sp*.		
ME90	*Alstonia scholaris*	Mangaluru	*Enterobacter sp*.		
ME91	*Alstonia scholaris*	Mangaluru	*Enterobacter sp*.		
ME92	*Alstonia scholaris*	Mangaluru	*Enterobacter sp*.		
ME93	*Alstonia scholaris*	Mangaluru	*Pseudomonas sp*.		
**ME94**	***Alstonia scholaris***	**Mangaluru**	***Methylobacterium sp*.**	***Methylobacterium radiotolerans***	***Methylobacterium radiotolerans***
ME95	*Alstonia scholaris*	Mangaluru	*Pseudomonas sp*.		

a Genome sequenced medicinal plant endophytes (ME) are highlighted in bold font

Bacterial 16S rRNA gene sequences were analysed using Nucleotide BLAST implemented on the NCBI server (https://blast.ncbi.nlm.nih.gov) against the nucleotide collection (nr/nt) and the 16S ribosomal RNA sequences databases to identify closest relatives. Sequences were assigned to various operational taxonomic units (OTUs) by using BLASTClust (http://www.ncbi.nlm.nih.gov/) at 95% similarity, representing a genus level grouping (Schloss and Handelsman 2004). Diversity measurements including rarefaction curves, coverage, Shannon's and Simpson's indices of diversity and species richness (*S*_Chao1_) were calculated using the Past software package v3.14 (Hammer, Harper and Ryan 2001).

All 16S rRNA gene sequences were aligned using MAFFT v7 online (Katoh, Rozewicki and Yamada 2019) with sequences retrieved from the database. Alignments were edited manually using BioEdit (Hall 1999) and phylogenetic trees were constructed using MEGA7 (Kumar, Stecher and Tamura 2016) by using the Maximum Likelihood method with the General Time Reversible model and Gamma distribution. Congruent trees were also obtained using other methods, including minimum evolution and LogDet distance, neighbour-joining with Jukes-Cantor algorithm.

### Bacterial genome sequencing and assembly

Bacterial genomic DNA was extracted from isolates of interest (*n* = 26), identified by 16S rRNA gene sequencing, from a 3 mL overnight culture grown in TSB or R2A at 30°C. Cells were collected by centrifugation at 4000 rpm using ALC PK120 Centrifuge for 10 min, resuspended in 4M guanidinium Isothiocyanate and DNA extracted using an automated Maxwell® 16 Instrument with Tissue DNA Purification Kits (Promega UK Ltd, Southampton, UK) according to the manufacturer's instructions. DNA was quantified using a Qubit 3.0 Fluorometer, and libraries prepared for 250 bp nucleotide paired-end sequencing using the NEBNext® Ultra II DNA Library Prep Kit for Illumina. Genome libraries were then sequenced by an Illumina MiSeq platform.

Sequence reads were trimmed from Illumina adaptors using the TrimGalore v0.4.2 script (https://www.bioinformatics.babraham.ac.uk/projects/trim_galore/) and paired reads were merged with FLASH v1.2.11 (Magoc and Salzberg 2011). Genomes were assembled with SPAdes v3.13.0, and mis-assemblies corrected using Pilon v1.22 (Bankevich *et al*. 2012; Walker *et al*. 2014).

Bacterial genome and 16S rRNA gene sequences reported in this study have been submitted to the European Nucleotide Archive (ENA) under the project/study accession number PRJEB37902.

### Species Identification of bacterial endophyte genomes

To allow species identification of the 26 bacterial endophyte genomes, the genus of each bacterial isolate was initially assigned by 16S rRNA gene comparison to the NCBI BLAST database coupled with genome identification using the taxonomic sequence classification system Kraken2 v2.0.6-beta and RefSeq complete bacterial genomes. Using this preliminary identification as a guide, full species assignment was then achieved by combining average nucleotide identity (ANI) and core-gene phylogenomics. The MinHash-based ANI tool FastANI (Jain *et al*. 2018) was used to identify RefSeq genomes of the same genus with high sequence similarity to each endophyte isolate. RefSeq genomes of each genus were downloaded using a NCBI genome download script available at GitHub (https://github.com/kblin/ncbi-genome-download). Up to 30 genomes with >90% sequence identity to each isolate, in addition to other endophyte isolates of the same genus, were passed to an alignment-based ANI tool, PyANI (Pritchard *et al*. 2016) for enhanced ANI accuracy. A core-gene phylogeny was constructed for each genus comprising genomes (Refseq and endophyte isolates) with >95% sequence identity to a given isolate, in addition to type strains and additional species representatives. Core-gene alignments were generated with Roary v3.13.0 (Page *et al*. 2015) implementing MAFFT v7.407 (Katoh and Standley 2013) and using genome annotations produced with Prokka v1.12. Maximum-likelihood phylogenetic trees were constructed using RaxML v8.2.12 with a general time reversible substitution model and gamma model of rate heterogeneity; and visualised with FigTree (http://tree.bio.ed.ac.uk/software/). In addition, for comparison genome sequences were also uploaded to the Type (strain) Genome Server (TYGS) bioinformatics platform available (https://tygs.dsmz.de) for whole genome-based taxonomic analysis (Meier-Kolthoff and Göker 2019). This platform provides both species assignment and digital DNA–DNA hybridisation (dDDH) values to the closest type strain genomes available.

### Assessing biosynthetic gene cluster potential of whole-genome sequenced endophytes

To ascertain the biosynthetic gene cluster (BGC) potential of bacterial endophytes the genomes were analysed with the specialised metabolite predicting software antiSMASH v4.0 (Blin *et al*. 2017). Following BGC prediction, the sequences were extracted for de-replication to understand the overall biosynthetic diversity of the endophyte genome collection. BGC sequences were grouped according to genus and de-replicated using a pairwise k-mer-based comparison with Mash v2.2 (Ondov *et al*. 2016) and applying a maximum distance threshold of 0.24. De-replication was performed with the assumption that BGCs would not be shared between the different genera. The resulting distance network was visualised using Cytoscape v3.4.0 (Shannon 2003). Manual curation of the network was required to identify instances of BGCs split across multiple contigs and erroneously predicted hybrid BGCs due to close genomic locus proximity.

### *In vitro* antagonism assays

Genome-sequenced medicinal plant endophytes (ME) were tested for antimicrobial activity against a small panel of human and plant pathogens (*Pectobacterium carotovorum* LMG 2464; *Staphylococcus aureus* NCTC 12981; *Candida albicans* SC5314) using an agar overlay inhibition assay as described (Mullins *et al*. 2019). In brief, ME isolates were grown overnight at 30°C on agar-solidified basal salts medium supplemented with glycerol (BSMG). After 24 h growth, a 10 µL-sized loopful of bacteria was resuspended in 1 mL phosphate buffered saline (PBS) buffer, spotted (3 µL volume) onto BSMG plates and incubated at 30°C for 48 h. ME isolates were killed by chloroform exposure for 2 mins, overlaid with pathogen-seeded half-strength iso-sensitest agar (Oxoid) supplemented with 0.2% (w/v) triphenyl tetrazolium chloride and incubated at 30°C or 37°C for 24 h.

## RESULTS

### Medicinal plants from the Western Ghats contain high diversity of bacterial endophytes

A total of 26 different medicinal plant samples (Table S1 and Figure S1a, Supporting Information) were taken from two sites in the Western Ghats and one site in Mysore, India. This represented one of the largest surveys of bacterial endophytes in Indian plants used for multiple medicinal purposes (Table S1, Supporting Information). The incubation of inoculated leaf tissue samples on LB agar readily enabled the growth of culturable endophytes from medicinal plants (Figure S1b, Supporting Information). During incubation, visible colonies were easily distinguishable on the edges of the leaf sections. After further subculture and incubation, 95 plant endophyte cultures were collected. These cultures were then further purified on TSA/R2A and checked for purity using 16S rRNA gene sequencing which resulted in 75 pure cultures of medicinal plant endophytes (designated as ME isolates). The assembled pure bacterial collection included 50 ME Gram-negative and 25 ME Gram-positive bacterial isolates (Table 1).

**Figure 1.**
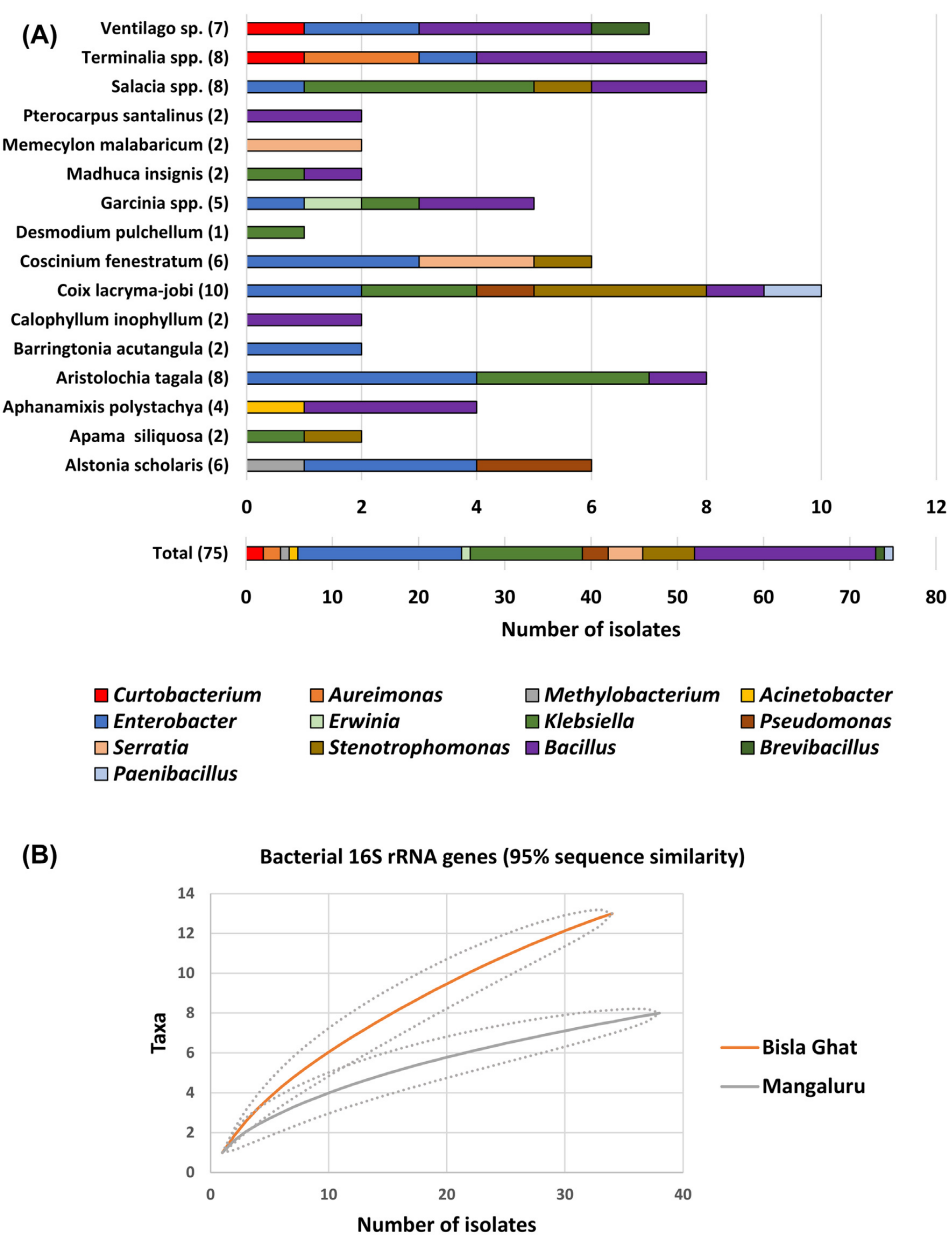
Community composition and rarefaction curves for the Western Ghats medicinal plant endophyte (ME) collection isolated during this study. **(A)** Composition of the bacterial endophyte collection assigned at the taxonomic genus level based on 16S rRNA genes. Each bar represents the relative distribution of each bacterial genus isolated from different medicinal plant genera. Numbers in parentheses represent the number of ME obtained from each plant genera. **(B)** Rarefaction curves for bacterial endophyte 16S rRNA gene diversity. ME were isolated from leaves of plants from the Bisle Ghat and Mangaluru regions of the Western Ghats, India. Curves were plotted for 95% similarity for 16S rRNA genes. Note, since there were only three isolates from Mysore leaf samples, this endophyte collection is not included.

Overall, from the three locations sampled (Bisle Ghat, Mysore and Mangaluru) pure culturable endophytes were isolated from 20 plant species covering 16 plant genera (Fig. 1A; Table 1). Only three plant genera (four plant species: *Nothapodytes nimmoniana, Garcinia gummi-gutta, Kingiodendron pinnatum and Dysoxylum binectariferum*) were unsuccessful in ME pure culture isolation. Interestingly, diversity indices and rarefaction analysis calculated at the bacterial genus level (Fig. 1B; Table 2; Figure S2, Supporting Information) suggested that the endophyte population collected from Bisle Ghat (34 isolates) was more diverse and species rich than the populations collected at Mangaluru (38 isolates) or Mysore (three isolates). In addition, rarefaction curves (Figure S2, Supporting Information) and Good's coverage statistics (Table 2) suggest that the total culturable bacterial diversity has not yet been isolated from the medicinal plants investigated in this study and further analysis is necessary to identify the full range of bacterial endophytes present.

**Table 2.** Diversity indices for bacterial endophyte 16S rRNA gene sequences using genus-level groupings (95% similarity).

Diversity indices	All isolates	Bisle Ghat region	Mangaluru	Mysore
**Number of isolates**	75	34	38	3
**Unique OTUs**	16	13	8	2
**Good's coverage (%)**	79	62	79	33
**Simpson's diversity index (1-*D*)**	0.72	0.82	0.60	0.44
**Shannon's diversity index (*H*')**	1.88	2.10	1.33	0.64
***S*_Chao1_**	23	17	11	2

OTU, operational taxonomic unit.

*S*_Chao1_ represent the expected number of OTUs present in an environment if sampling were complete.

Shannon's and Simpson's indices are measures of species diversity and both increase with increasing genetic diversity.

Using 16S rRNA gene sequence similarity all medicinal plant endophytes were representatives of three bacterial phyla (Proteobacteria, 66%; Firmicutes 31%; Actinobacteria, 3%; Fig. 2; Figure S3, Supporting Information) belonging to the following 13 genera (Fig. 1A) in order of dominance: *Bacillus*, 28%; *Enterobacter*, 25.4%; *Klebsiella*, 17.3%; *Stenotrophomonas*, 8%; *Serratia*, 5.4%; *Pseudomonas*, 4%; *Acinetobacter*, 1.3%; *Aureimonas*, 1.3%; *Curtobacterium*, 1.3%; *Brevibacillus*, 1.3%; *Erwinia/Pantoea*, 1.3%; *Methylobacterium*, 1.3%; *Paenibacillus*, 1.3%.

**Figure 2.**
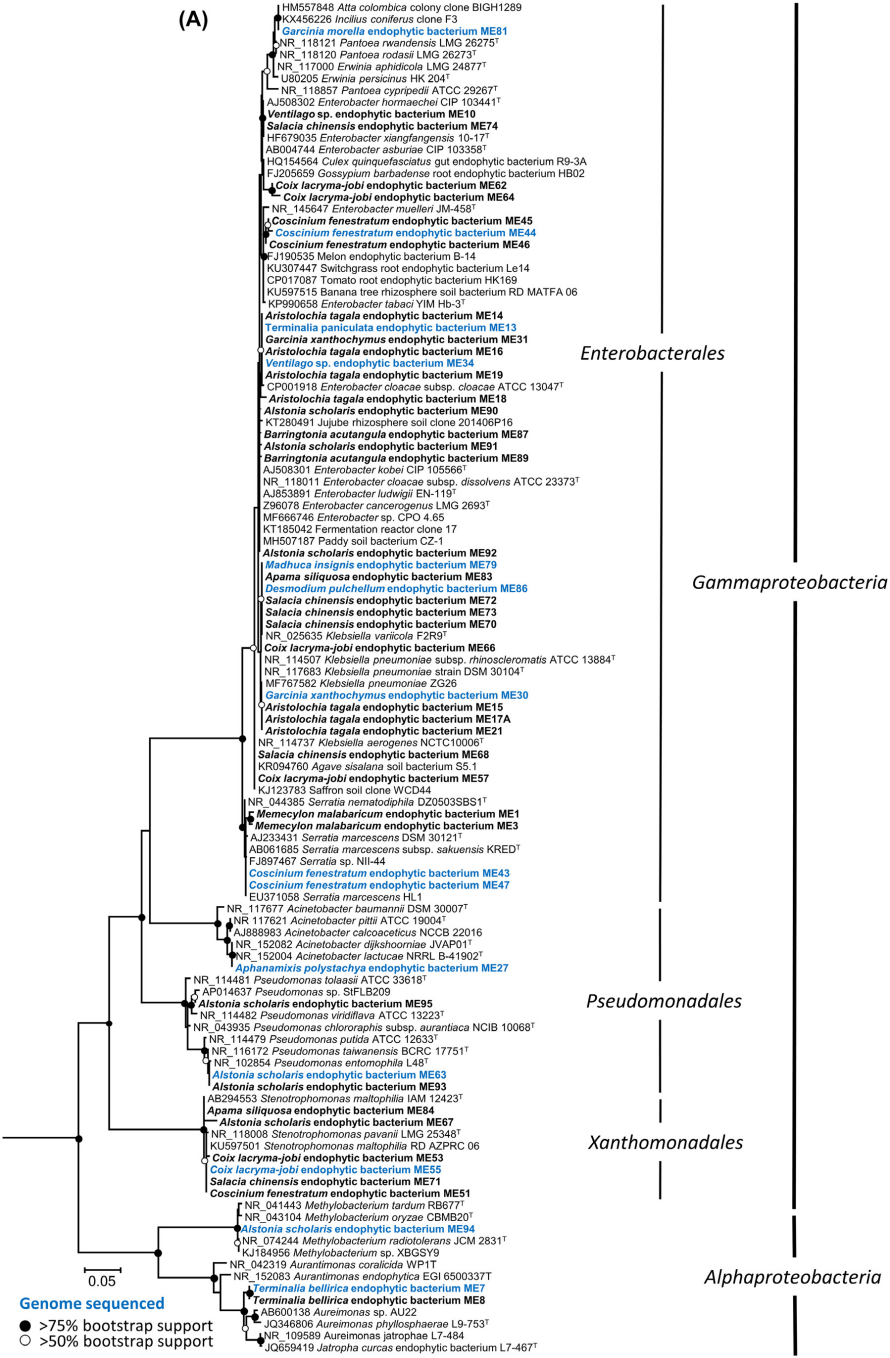
Phylogenetic trees showing the relationship of medicinal plant endophyte (ME) 16S rRNA gene sequences to sequences from representative type species and other plant endophytes. **(A)***Proteobacteria* (Gram-negative) and **(B)***Firmicutes* and *Actinobacteria* (Gram-positive). Trees were constructed using Maximum Likelihood method based on the GTR model. A discrete Gamma distribution was used to model evolutionary rate differences among sites. All positions containing gaps and missing data were eliminated and there was a total of 627 and 695 positions in the final datasets, respectively. *Deltaproteobacteria* 16S rRNA gene sequences were used as outgroups in (A) *Desulfobacter curvatus* DSM 3379 (AF418175), *Desulfuromonas acetoxidans* DSM 684 (AAEW02000008), *Desulfovibrio aerotolerans* Dv06 (AY746987); and *Proteobacteria* 16S rRNA gene sequences were used as outgroups in (B) *Methylobacterium radiotolerans* JSCM 2831 ( NR_074244), *Desulfovibrio aerotolerans* Dv06 (AY746987), *Enterobacter ludwigii* EN-119^T^ (AJ853891). Evolutionary analyses were conducted in MEGA7. The percentage of trees in which the associated taxa clustered together is shown next to the branches, based on 100 bootstraps. Nodes with black circles represent >75% bootstrap support; nodes with white circles represent >50% bootstrap support. The scale bar represents 5% sequence divergence. Sequences in bold represent ME isolates and sequences in bold blue represent ME isolates that had their genomes sequenced.

**Figure 2.**
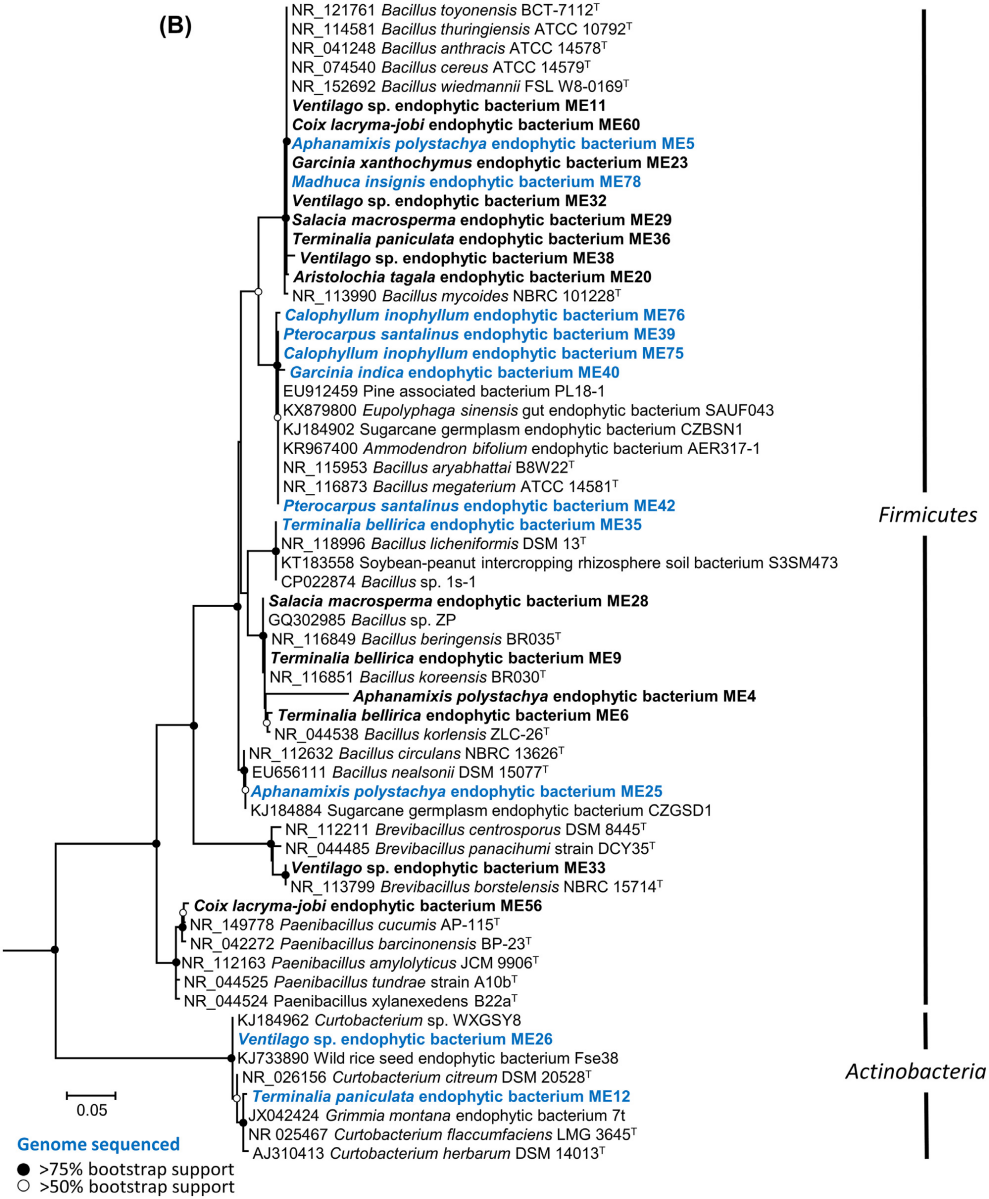
continued

The grass species *Coix lacryma-jobi* was observed to contain the highest culturable diversity (ten isolates) of MEs (Fig. 1A; Table 1) with six different bacterial genera present (*Enterobacter, Klebsiella, Pseudomonas, Stenotrophomonas, Bacillus and Paenibacillus*). Contrastingly, the shrub *Desmodium pulchellum* had the lowest culturable diversity with only one bacterial isolate belonging to *Klebsiella* (ME86). Both plants were sampled from the Mangaluru location. For comparison, *Aristolochia tagala*, a climbing species found in forests of Asia had the highest culturable diversity (eight isolates) of MEs (*Enterobacter, Klebsiella* and *Bacillus*) identified from leaf samples taken from the Bisle Ghat (Fig. 1A; Table 1).

Multiple ME isolates were taxonomically related (based on 16S rRNA gene similarity) to previously known endophytes or bacteria isolated from soil and rhizosphere environments (Fig. 2). For example, the large collection of ME *Enterobacter* (19 isolates) are closely related (Fig. 2A) to endophytes from tomato, switchgrass, banana, jujube, cotton and rice paddy soils, and were isolated from a range of medicinal plants which include *Ventilago* sp., *Salacia chinensis*, *Coix lacryma-jobi*, *Coscinium fenestratum*, *Aristolochia tagala*, *Terminalia paniculata*, *Garcinia xanthochymus*, *Alstonia scholaris* and *Barringtonia acutangular* sampled from both the Bisle Ghat and Mangalaru locations. Similarly, the 21 ME isolates belonging to the genus *Bacillus* (12 different plant species and 3 locations) are related to endophytes from pine trees, sugar cane, legumes as well as insect guts (Fig. 2B). Many of these *Bacillus* isolates (ten isolates) were closely related to soil-borne bacteria within the *Bacillus cereus* group (Rasko *et al*. 2005; Carroll, Wiedmann and Kovac 2020).

### Average nucleotide identity and dDDH reveal the predominant bacteria in the sequenced panel as *Bacillus spp*. and *Enterobacteriaceae*

**Figure 3.**
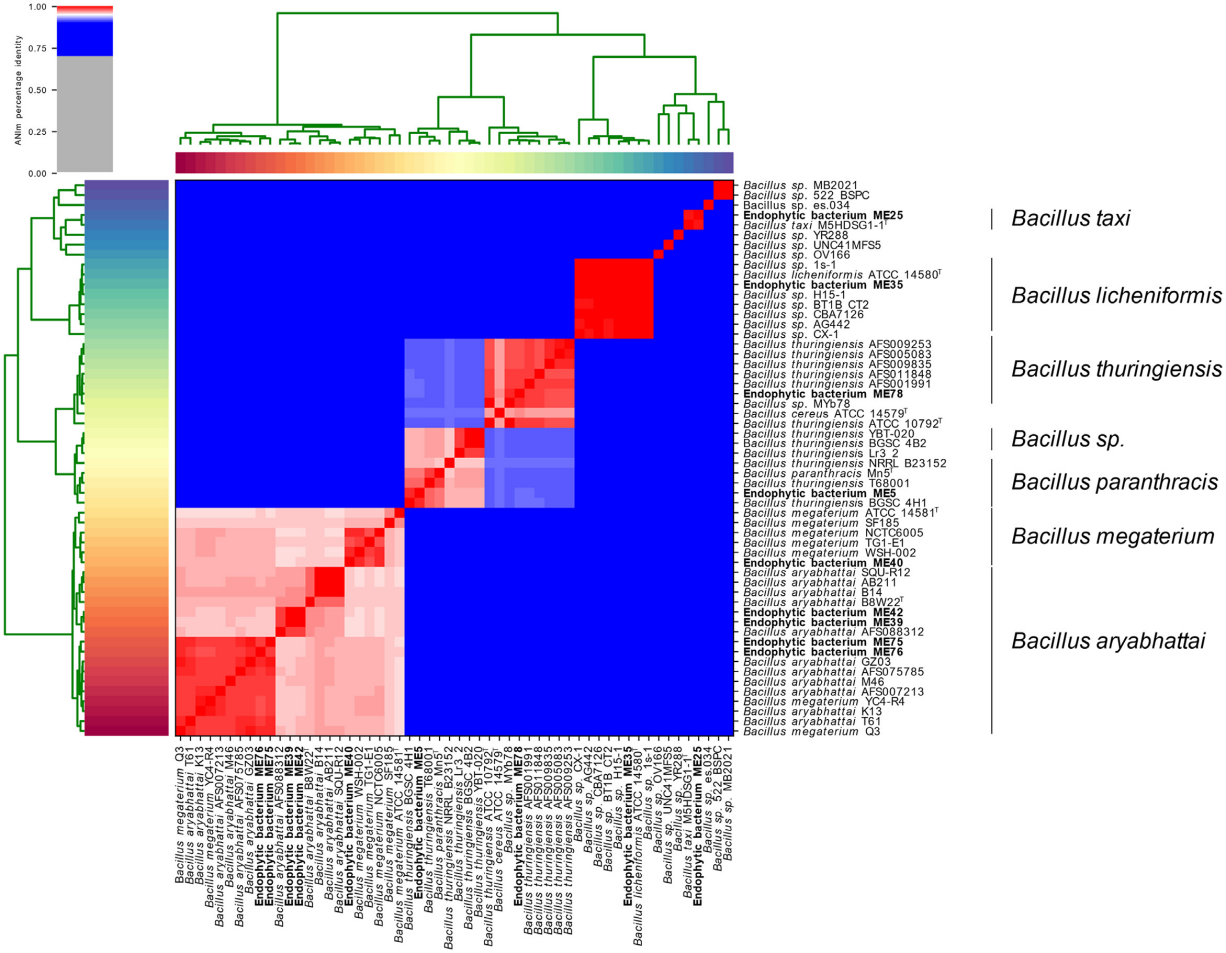
Genome sequence taxonomic placement of *Bacillus* medicinal plant endophytes (ME) inferred by average nucleotide identity (ANI). Heatmap generated by the PyANI script, indicating the degree of nucleotide-level similarity between *Bacillus* species ME and their closest reference strains. ME are highlighted in bold font, whilst species type strains are denoted by ^T^. Colour indicates the degree of nucleotide similarity, with red areas indicating >95% ANI, and darker shades of red indicating greater similarity. Blue indicates <95% ANI.

**Figure 4.**
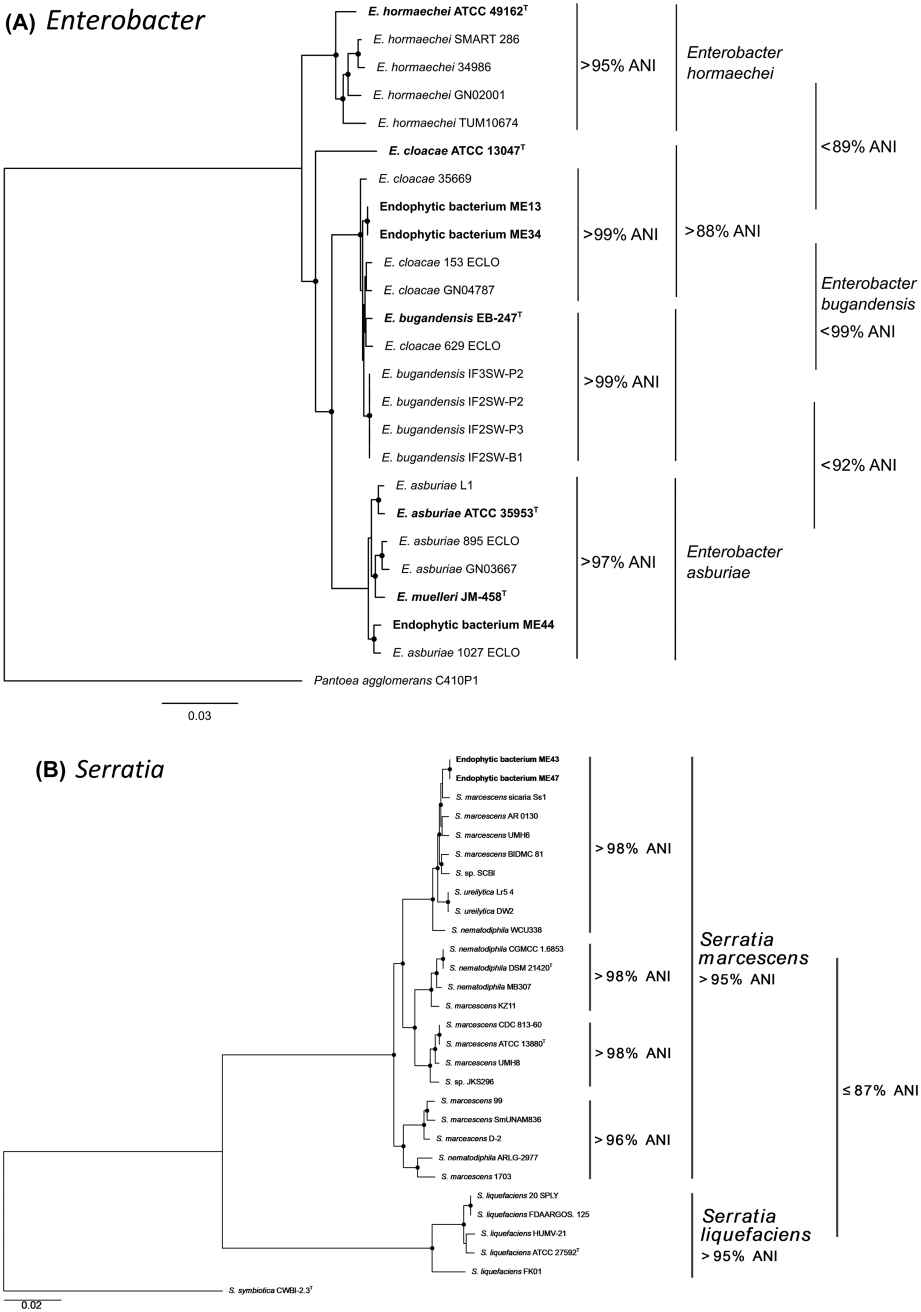
Average nucleotide identity (ANI) and core genome analysis of medicinal plant endophytes (ME) belonging to the order Enterobacterales: **(A)***Enterobacter* and **(B)***Serratia*. (A) Core-gene phylogeny of ME belonging to *Enterobacter* species. A 1912 core-gene alignment generated by Roary was used to construct a maximum likelihood tree highlighting the placement of *Enterobacter* endophytes. Isolates ME13 and ME34 placed within the *E. cloacae* species clade, whilst ME44 placed within the *E. asburiae* clade. (B) Core-gene phylogeny of ME belonging to *Serratia* species. A 255 core-gene alignment generated by Roary was used to construct a maximum likelihood phylogeny highlighting the placement of isolated *Serratia* endophytic bacteria. ME43 and ME47 were placed within the *Serratia marcescens* species clade with >95% ANI to other members of the species. Phylogenetic trees were constructed with GTR model with gamma substitution and supported by 100 bootstraps. Nodes with black circles represent >90% bootstrap support. Scale bar = substitutions per site.

**Figure 5.**
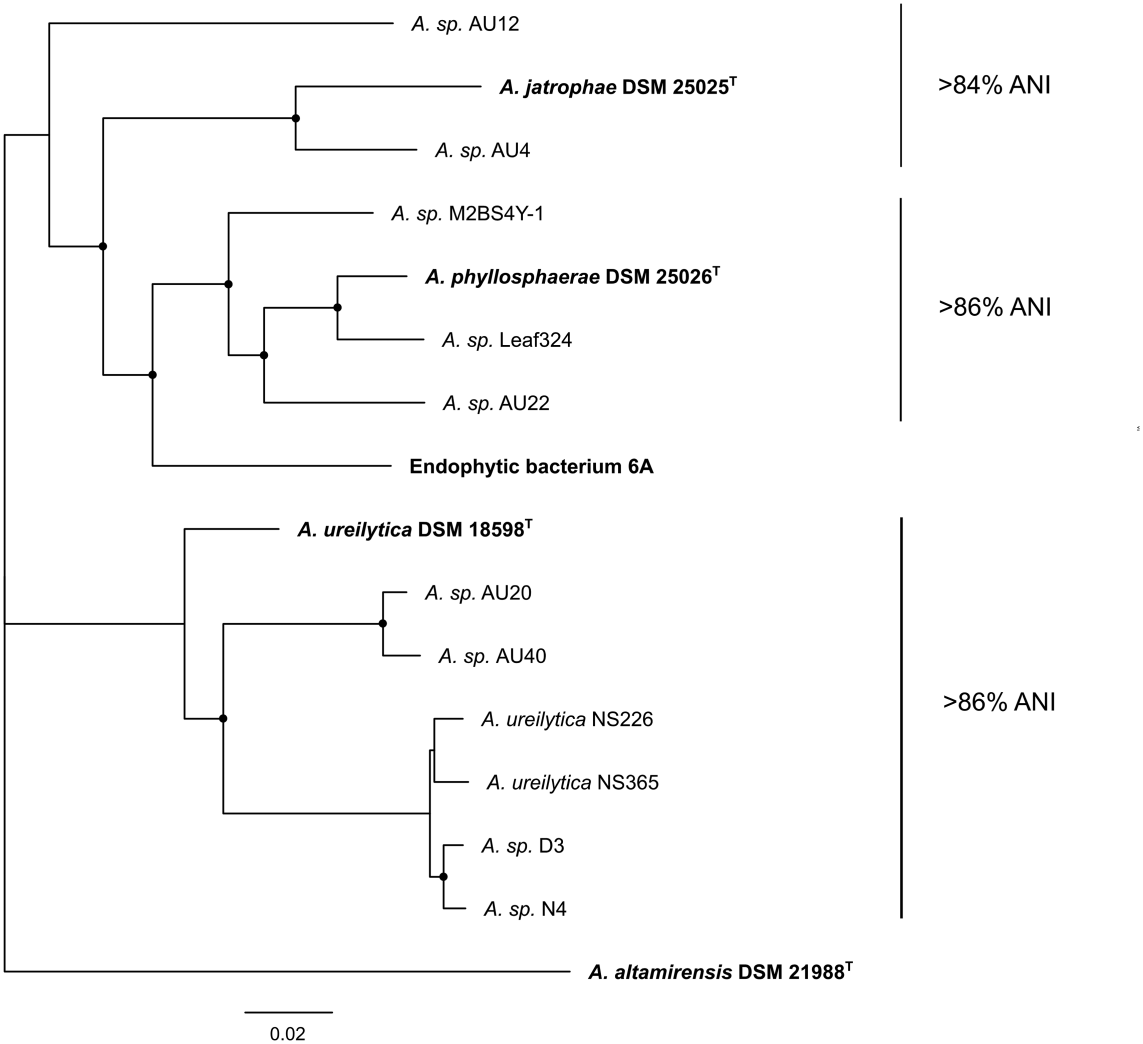
Core-gene phylogeny of medicinal plant endophytes (ME) belonging to *Aureimonas* species. A 25 core-gene alignment generated by Roary was used to construct a maximum likelihood tree highlighting the placement of *Aureimonas* endophytes. Isolate ME7 was placed as a novel species close to the *A. phyllosphaerae* clade. The phylogenetic tree was constructed with GTR model with gamma substitution and supported by 100 bootstraps. Nodes with black circles represent >90% bootstrap support. Scale bar = substitutions per site.

Few endophytes have been genome sequenced as part of their collection and initial characterisation. A total of 26 selected medical plant endophytes were genome sequenced to increase the level to which they could be identified and characterised. The genus-level diversity of the genome sequenced endophytes as determined by genomic ANI and dDDH analysis (Table 1) were as follows: *Bacillus* (*n* = 9), *Klebsiella* (*n* = 4), *Enterobacter* (*n* = 3), *Curtobacterium* (*n* = 2), Serratia (*n* = 2), *Aureimonas* (*n* = 1), *Stenotrophomonas* (*n* = 1), *Acinetobacter* (*n* = 1), *Pantoea* (*n* = 1), *Methylobacterium* (*n* = 1) and *Pseudomonas* (*n* = 1). A summary of the genome assembly metrics is given in Table S2 (Supporting Information). Overall identification at the genus-level by genome analysis was in agreement with classification by 16S rRNA gene identification (Figs 2–5, Table 1, Figures S4–S9, Supporting Information) with the exception of isolate ME81 which was identified initially by 16S rRNA gene analysis as *Erwinia* (Fig. 2A) but subsequently classified by both genome analysis methods as *Pantoea* (Table 1, Figure S7, Supporting Information).

The species-level identity provided was consistent between ANI and dDDH for all isolates, excluding five *Bacilli*. A total of two *Bacillus* isolates, ME5 and ME78, were identified as *Bacillus thuringiensis* (98.8%) by ANI but were identified as *Bacillus paranthracis* and *Bacillus cereus* respectively by dDDH using TYGS. The remaining isolates, ME40, ME75 and ME76 could not be assigned a species-level identity by TYGS, but were identified as *Bacillus megaterium* (ME40, 95.8% identity), and *Bacillus aryabhattai* (ME75 and ME76, 96.3% identity) respectively by ANI. A heatmap providing a visual representation of the full diversity of *Bacillus* endophytes by ANI is shown in Fig. 3.

### Core-gene analysis indicates a high-degree of intra-genus similarity for *Enterobacter* and *Serratia* endophytes, whilst highlighting the novelty of the *Aureimonas sp*. ME7

To increase the resolution of genomic taxonomy applied to the endophytic bacterial collection phylogenomic approaches were also applied on selected genera as follows. Core-gene phylogenetic analysis (Fig. 4) revealed three genera of interest, due to either the high degree of similarity between one or more sequenced endophytes (*Enterobacter* and *Serratia*) or unique phylogenetic placement supported by ANI, indicating a novel species group (*Aureimonas*). The analysis of *Enterobacter* genomes revealed that endophyte ME13, isolated from *Terminalia paniculata* in the Bisle Ghat region possessed the same core-genes as endophyte ME34, isolated from *Ventilago* sp. in the same region (Fig. 4A). The nearest neighbour of these isolates was the genome of *Enterobacter cloacae* 153_ECLO. This isolate, and both ME13 and ME34, were however distinct from the *E. cloacae* type strain, ATCC 13047^T^, both phylogenetically and in terms of ANI. Core-gene analysis of *Serratia* genomes revealed that endophytes ME43 and ME47, both of which were isolated from *Coscinium fenestratum* in the Mangalaru region were identical in terms of core-gene content (Fig. 4B). Additionally, both ME43 and ME47 possessed ≥95% identity in comparison to the *Serratia marcescens* type strain, ATCC 13880^T^ (Fig. 4B).

Notably, core-gene analysis revealed *Aureimonas* sp. isolate ME7 as a novel endophyte, as shown by its unique phylogenetic placement (Fig. 5). All sequenced genomes obtained for the genus *Aureimonas* were extremely diverse, displaying deep phylogenetic branching and ANI values far below the established 95% threshold for species delineation (≥85% identity). The nearest neighbours for this isolate were all known endophytes and included *Aureimonas sp*. AU22 and Leaf324 from soybean and *Arabidopsis thaliana*, respectively.

Core-gene phylogenetic analyses for endophytes that did not belonging to genera of interest can be found in Figures S4–S9 (Supporting Information). In addition, since only a limited number of genomes are available for the Actinobacteria genus, *Curtobacterium* full-length 16S rRNA gene phylogenies were constructed instead (Figure S8, Supporting Information). Phylogenetic analysis demonstrated that endophyte ME12 (from *Terminalia paniculate*) was closely related (99% sequence similarity) to the plant pathogen *Curtobacterium flaccumfaciens* strains and that isolate ME26 from *Ventilago* sp. was related (99% sequence similarity) to novel endophytic *Curtobacterium* sp. WXGSY8 from sugarcane and *Curtobacterium* sp. ER1/6 from *Citreus sinensis* (sweet orange), a potential biocontrol strain (Garrido *et al*.^2016^). The frequent isolation of *Curtobacterium* as endophytes from asymptomatic citrus plants infested with the pathogen *Xylella fastidiosa* indicated that endophytic *Curtobacterium* species may help to resist infection (Rosenblueth and Martínez-Romero 2006).

### Biosynthetic gene cluster prediction revealed both known and uncharacterised specialised metabolites

Following the prediction and curation of BGCs of the 26 sequenced endophyte genomes, a total of 102 distinct BGCs were identified across the 11 bacterial genera. These BGCs represented 15 known metabolite classes including siderophores, lassopeptides and non-ribosomal peptides (Table 3). Approximately 15% of BGCs could not be assigned a class and were collated under the antiSMASH category ‘Other’. The most prevalent classes were non-ribosomal peptides synthetases (NRPS), terpenes and bacteriocins representing approximately 45% of curated BGCs. The genus *Bacillus* with nine ME isolates contributed the majority of predicted BGCs to the endophyte biosynthetic potential, representing one-third of the 102 gene clusters (Table 3).

**Table 3.** Summary of biosynthetic gene cluster (BGC) potential of medicinal plant endophytic bacteria. AntiSMASH was used to predict BGCs in the 26 whole-genome sequenced endophytic isolates. The predicted BGCS were curated and de-replicated to determine the number of distinct BGCs in the genome collection.

				Specialised metabolite class															
Genus	Number of genomes	AntiSMASH predicted BGCs	Distinct BGCs	NRPS	Bacteriocin	T3PKS	NRPS-T1PKS	T1PKS	Thiopeptide	Lassopeptide	Lantipeptide	Terpene	Phosphonate	Arylpolyene	Butyrolactone	Homoserine lactone	Microcin	Siderophore	Other
																			
*Bacillus*	9	67	34	7	5	1	1	0	1	2	5	5	1	0	0	0	0	3	3
*Klebsiella*	4	17	6	1	1	0	1	0	1	0	0	0	0	0	1	0	1	0	0
*Enterobacter*	3	15	6	1	0	0	0	0	1	0	0	0	0	1	1	1	0	1	0
*Serratia*	2	33	10	4	1	1	2	0	1	0	0	0	0	0	0	1	0	0	0
*Curtobacterium*	2	12	8	0	0	2	0	0	0	0	0	1	0	0	0	0	0	1	4
*Acinetobacter*	1	6	7	2	1	0	0	0	0	0	0	0	0	2	0	1	0	1	0
*Aureimonas*	1	4	4	0	0	0	0	1	1	0	0	2	0	0	0	0	0	0	0
*Methylobacterium*	1	8	8	0	0	0	0	1	0	0	0	3	0	0	0	2	0	0	2
*Pantoea*	1	11	9	2	1	0	0	0	1	0	0	2	0	2	0	1	0	0	0
*Pseudomonas*	1	18	6	2	1	0	0	0	0	0	0	0	0	0	1	1	0	0	1
*Stenotrophomonas*	1	4	4	1	2	0	0	0	0	0	0	0	0	1	0	0	0	0	0
																			
Total	26	195	102	20	12	4	4	2	6	2	5	13	1	6	3	7	1	6	10

BGC, biosynthetic gene cluster; NRPS, nonribosomal peptide synthase; T1PKS, Type 1 polyketide synthase; T3PKS, Type 3 polyketide synthase; NRPS-T1PKS, nonribosomal peptide synthase-Type 1 polyketide synthase hybrid

Only four hybrid non-ribosomal peptide synthetase-polyketide synthases (NRPS-PKS) were predicted, one of the hybrid BGCs from *Klebsiella* sp. ME86 possessed similarity to the yersiniabactin BGC, while the remaining three of these represented uncharacterised BGCs. Additional known BGCs identified in the endophyte genomes included the lassopeptide genes responsible for lichenicidin synthesis in *Bacillus* sp. ME35, and the NRPS required for acinetobactin synthesis in *Acinetobacter* sp. ME27. The majority of endophyte derived BGCs lacked homology to known specialised metabolite BGCs using the MiBIG database (Medema *et al*. 2015) via antiSMASH (Blin *et al*. 2017) that was applied.

### Antimicrobial activity of medicinal plant endophytes

A total of five of the 26 genome-sequenced bacterial endophytes showed antimicrobial activity against the plant pathogen, *Pectobacterium carotovorum* (Figure S10, Supporting Information for examples). However, no zones of clearing were observed for the pathogens *Staphylococcus aureus* or *Candida albicans* by any ME tested. Isolates with clear antibacterial activity against the Gram-negative bacterium, *P. carotovorum* were identified as *Bacillus aryabhattai* (ME39), *Bacillus* sp. (ME40), *Enterobacter asburiae* (ME44) and *Serratia* sp. (ME43 and ME47). A total of four additional isolates showed weak antimicrobial activity against *P. carotovorum*: *Bacillus aryabhattai* (ME42), *Bacillus* sp. (ME75), *Klebsiella pneumoniae* (ME30) and *Klebsiella variicola* (ME73) (Figure S10, Supporting Information). Interestingly, several of the ME isolates with antibacterial activity and with predicted BGCs were obtained from medicinal plants used in traditional medicine for the treatment of wounds and/or known to have described antimicrobial activity. For example, *Coscinium fenestratum* (isolates ME43, ME44 and ME47) and *Garcinia* species (isolates ME30 and ME40) plant extracts have shown activity against *Escherichia coli* and other pathogenic bacteria (Nair *et al*. 2005; Baliga *et al*. 2011; Joseph, Dandin and Murthy Hosakatte 2016).

## DISCUSSION

### Medicinal plant endophyte diversity

Using a cultivation-based approach we have successfully isolated and identified 75 fast-growing cultivable bacteria that were associated with leaves of different plant species. Previously, endophytes have been reported from various other traditional medicinal plants; for example, *Gynura procumbens* (Bhore, Nithya and Loh 2010), *Artemisia annua* (Li *et al*. 2011), *Tridax procumbens* (Preveena and Bhore 2013), ginseng (Khan Chowdhury *et al*. 2017) and other traditional Chinese herbs (Miller *et al*. 2012a). However, to our knowledge, this study is unique in exploring a diverse range of bacterial isolates from a large collection (covering 24 plant species) of medicinal plants from the Western Ghats region of India. The identified bacterial endophytes belonged to four major taxa, Alphaproteobacteria, Gammaproteobacteria, Actinobacteria and Firmicutes, with isolates from the following genera: *Bacillus, Enterobacter, Klebsiella, Stenotrophomonas, Serratia, Pseudomonas, Acinetobacter, Aureimonas, Curtobacterium, Brevibacillus, Pantoea, Methylobacterium* and *Paenibacillus*. Previously, culture-based bacterial endophyte diversity analysis has shown that most culturable endophytes are Proteobacteria, followed by Actinobacteria, Bacteroidetes and Firmicutes (Rosenblueth and Martínez-Romero 2006; Khan Chowdhury *et al*. 2017). The same limited group of bacterial phyla were also found to predominate in the phyllosphere of different plants identified by a range of culture-independent approaches including metagenomic shotgun sequencing of total genomic DNA (Vorholt 2012). However, concerted efforts have been made to study Actinobacteria since they are a major source of natural antibiotics and metabolites (Passari *et al*. 2017; Ek-Ramos *et al*. 2019), while other bacterial phyla are a natural resource that are still relatively untapped.

A previous study observed that higher culturable endophytic bacterial diversity was associated with a higher likelihood of the host plant exhibiting antimicrobial properties (Egamberdieva *et al*. 2017). However, in contrast to bacterial diversity, this study also reported that the total bacterial cell numbers of colonizing microbes maybe higher in plants that have poor antimicrobial activity (Egamberdieva *et al*. 2017). Presumably, this is due to less stringent conditions encountered in these plants which allows for high numbers of colonizing bacteria to proliferate due to the lack of competition and lower concentrations of antimicrobials. In our study, only a tentative relationship was observed, linking high culturable microbial diversity to previously known medicinal properties for the treatment of bacteria-associated disease or known antibacterial activity. The medicinal plants with the highest culturable bacterial diversity, *Terminalia* spp., *Ventilago* sp. and *Salacia* spp. are used to treat bacterial diseases and leaf extracts of *Coix lacryma-jobi* and *Coscinium fenestratum* (Nair *et al*. 2005; Das *et al*. 2017) have been reported to have antimicrobial properties. However, we also observed plants with similar medicinal uses to have a low culturable diversity of ME isolates, namely *Calophyllum inophyllum* and *Memecylon malabaricum* (Table S1, Supporting Information). It should be noted that total bacterial numbers found within the leaves were not counted in our study. Further studies may be necessary to address the issue of achieving full culturable diversity, through focused efforts to isolate and count other endophytic community members including slow growing bacteria and fungi through the use of less complex and/or specific media (Eevers *et al*. 2015; Martinez-Klimova, Rodríguez-Peña and Sánchez 2017).

### The nearest neighbours to endophytes of interest can be isolated from a variety of environments

Core-gene analysis demonstrates that the nearest neighbours of all endophytes in this study are not limited to association with plants, but are instead ubiquitous, and able to endure a plethora of environments. This is evidenced in the analysis of *Enterobacter*, where the nearest neighbours to endophytes ME13 and ME34 (Fig. 4A), namely *E. cloacae* 35 669 (Doijad *et al*. 2016), 153_ECLO, 629_ECLO and GN04787 (Matteoli *et al*. 2020), were all isolated from clinical infections (Fig. 4A). In contrast, the nearest neighbours *Enterobacter bugandensis* IF2SW-B1, IF2SW-P2, IF2SW-P3 and IF3SW-P2 were all recently isolated from the International Space Station (Singh *et al*. 2018). The similarity of the space station isolates to clinical isolate 153 ECLO has been commented upon previously (Singh *et al*. 2018) and is thus concordant with the analysis in this study. Members of the *Enterobacter* genus also comprise species that have been reported as plant beneficial organisms and these include, plant-growth promoting endophytes of *Enterobacter asburiae* on date palm (Yaish 2016), *Enterobacter cloacae* with citrus and banana plants (Araujo *et al*. 2002; Macedo-Raygoza *et al*. 2019) and *Enterobacter* sp. J49, a biofertilizer for peanut and maize (Ludueña *et al*. 2019).

A number of nearest phylogenomic neighbours to *Serratia* endophytes ME43 and ME47, including *S. marcescens* AR_0130, BIDMC 81 and UMH6 (Anderson *et al*. 2017), originate from the nosocomial environment, whilst *S. marcescens* sicaria Ss1 was isolated from the haemolymph of worker bees suffering from sepsis and implicated as a new pathogen of honey bees (Burritt *et al*. 2016). However, isolates of *S. marcescens* are known to fix nitrogen and act as plant growth promoting endophytic colonisers of rice roots and stems (Gyaneshwar *et al*. 2001). Nonclinical isolates of *S. marcescens* have also been used as biocontrol agents (Hallmann *et al*. 1997) and induce systemic resistance to fungal and viral pathogens (Press *et al*. 1997), as well as the production of the biologically active compound prodigiosin (Khanam and Chandra 2018).

Interestingly, the *Aureimonas* endophyte ME7, was related to bacteria originally isolated from surfaces and internal tissues of plants and identified as a unique species by ANI and core-gene analyses (see Fig. 5). Members of the genus, *Aureimonas* are increasingly being isolated from leaves of plants (Madhaiyan *et al*. 2013; Li *et al*. 2017; Tuo and Yan 2019) and thought to be involved in the cycling of carbon and nitrogen (Ikeda *et al*. 2010). The nearest phylogenomic neighbours for this isolate were *Aureimonas* sp. AU22 and *Aureimona*s sp. Leaf324 isolated from the stems of soybean, and the leaves of *Arabidopsis thaliana* respectively, whilst the nearest neighbouring type-strains, *Aureimonas phyllosphaerae* DSM 25024^T^ and *Aureimonas jatrophae* DSM 25025^T^ were both isolated from the leaves of *Jatropha curcas* (Madhaiyan *et al*. 2013), a small tree whose seed oil is widely used as biofuel, soap and medicine (Pandey *et al*. 2012).

### Biosynthetic capacity of medicinal plant endophytes

Previous studies have investigated the NRPS and PKS diversity of medicinal plant bacterial and fungal endophytes through culture-independent PCR-based methods (Miller *et al*. 2012a). The benefits of this culture-independent approach included a lack of culture-bias and the ability to detect both fungal and bacterial NRPS and PKS potential. However, as noted, the limitations of a PCR screen were the inability to detect low level target DNA, and divergent sequence domains. Additional studies by Miller *et al*. (2012b) on Chinese medicinal plants obtained pure bacterial and fungal isolates that permitted cytotoxicity and antimicrobial phenotypic testing (Miller *et al*. 2012b). Although our culture-dependent isolation of endophytes was biased towards bacteria capable of growth on LB agar, the output of this study included draft whole-genome sequences and pure cultures of the isolated bacterial endophytes. This enabled both phenotypic testing of antimicrobial activity and genome mining for a multitude of biosynthetic gene clusters. A high proportion of the sequenced ME isolates possessed BGCs with NRPS and PKS-predictions (73% and 54%, respectively). This represents a significantly larger proportion than previously described endophyte collections (Miller *et al*. 2012b). However, this is partly biased by only examining the genome-sequenced portion of the collection, and the ability to predict type 3 polyketide synthase (T3PKS) and hybrid NRPS-PKS BGCs. The draft genomes described in our study enabled accurate taxonomic identification and resulted in the prediction of BGCs representing multiple metabolite classes, a contrast to existing work on medicinal plant endophytes.

The isolation of multiple endophytic bacterial isolates has previously been coupled to phenotypic assays of antimicrobial activity, tandem mass spectrometry analyses and PCR-based detection of conserved PKS and NRPS domains (Passari *et al*. 2017). This combinatory approach has led to promising leads of novel antimicrobial metabolites (Passari *et al*. 2017). Future work into the identification and isolation of metabolites of the endophyte collection in this study can be guided by the genomic insight into the biosynthetic origins of potential metabolites of these bacteria. Despite the identification of BGCs with high sequence similarity to previously characterised BGCs, most of the 102 biosynthetic gene clusters possessed no homology to published BGCs, and thus represent a novel source of pharmaceutically relevant products.

### Potential use of medicinal plant endophytes as antimicrobial and biocontrol agents

ME isolates which showed antibacterial activity towards the plant pathogen, *P. carotovorum* belonged to the genera *Bacillus* (*n* = 4), *Klebsiella* (*n* = 2), *Serratia* (*n* = 2) and *Enterobacter* (*n* = 1). Previously, *Bacillus* endophytes have demonstrated activity against bacterial phytopathogens (Ryan *et al*. 2008; Santoyo *et al*. 2016; Chen *et al*. 2019), including *P. carotovorum* (Wang *et al*. 2019). For example, the strain *Bacillus* sp. NA-HTong-7, isolated from the stems of the medicinal plant, *Dendrobium* possessed activity against both fungal (*Athelia rolfsii* and *Myrothecium roridum*) and bacterial (*P. carotovorum* subsp. *actinidiae*) pathogens of *Dendrobium* species and has potential as a biocontrol agent (Wang *et al*. 2019). Whereas, other studies have reported that *Bacillus* species isolated from medicinal plants exhibited general antibacterial activity including that against *Staphylococcus aureus, Escherichia coli*, *Klebsiella pneumoniae* and *Pseudomonas aeruginosa* (Akinsanya *et al*. 2015; Beiranvand *et al*. 2017; Egamberdieva *et al*. 2017) and suggest that they are responsible for the plants therapeutic properties. For a comprehensive review of the use of endophytes as therapeutic agents in Asian medicinal plants see the recent paper by Sharma and colleagues (Sharma *et al*. 2020).

*Bacillus* species have been found to be one of the most abundant metabolite-producing Gram-positive bacterial endophytes (Frank, Saldierna-Guzmán and Shay 2017), and in this study *Bacillus* were responsible for a third of all distinct BGCs identified (Table 3). *Bacillus* species produce a wide variety of antimicrobial metabolites, including ribosomally synthesised antimicrobial peptides (e.g. bacteriocins, lantipeptides and lassopeptides), as well as non-ribosomally synthesised peptides and polyketides (Zhao and Kuipers 2016). The *Bacillus* ME isolates (ME39 and ME40), with good bioactivity against *P. carotovorum* found in this study were shown by genome mining to contain several complete BGCs that may contribute to antimicrobial activity. *Bacillus aryabhattai* ME39 was shown to carry both lantipeptide and bacteriocin BGCs, while *Bacillus* sp. ME40 carried a lassopeptide BGC with sequence similarity and gene synteny to the paeninodin BGC. While the paeninodin lassopeptide lacked antimicrobial activity against representatives of Actinobacteria, Firmicutes and Proteobacteria (Zhu *et al*. 2016), antagonism against *P. carotovorum* was not investigated. However, several endophytic bacterial peptides with antimicrobial activity have been reported (Zhao and Kuipers 2016).

Other BGCs of interest in the remaining isolates with clear antagonism included hybrid NRPS-PKS BGCs in *Serratia* sp. ME47 with no homology to characterised BGCs; and thiopeptide BGCs predicted in *Enterobacter asburiae* ME44, *Serratias* isolates ME43 and ME47. Most characterised thiopeptides display nanomolar potency toward Gram-positive bacteria by blocking protein translation, and the majority of them have been identified from Actinobacteria and Bacilli (Schwalen *et al*. 2018). However, thiopeptides were also identified by genome mining in Proteobacteria (Schwalen *et al*. 2018). Our study shows the potential of *Bacillus* and other bacterial endophytes as biological control agents of plant pathogenic bacteria, and that ME isolates could be used to produce peptide-based antimicrobial and/or other compounds for therapeutic use. In addition, this study adds support to the claims and reports that some species of medicinal plants of the Western Ghats possess antimicrobial properties and may explain their ethnomedicinal use.

## CONCLUSIONS

This study identified multiple bacterial endophytes across a diverse array of medicinal plants from one of the World's ‘Hottest Hotspots’ of biodiversity, the Western Ghats (Myers *et al*. 2000). The draft genome assemblies obtained from these endophytes have permitted an insight into the biosynthetic diversity of these bacteria, whilst the isolation of pure cultures enables the future exploitation of the identified biosynthetic potential. To our knowledge, this represents one of the largest collections of isolates with draft genomes available from endophytic bacteria in a single study of medicinal plants. Identifying and understanding the medicinal plant endophytic microbial diversity therefore has potential for the discovery of new natural products.

## Supplementary Material

fiaa147_Supplemental_Files

## Notes

Department of Studies in Microbiology, University of Mysore, Mysore, Karnataka, 570006, India. Tel: +91 821 2419441; E-mail: raivittal@gmail.com
