# Supplementary material for: Culturable diversity of bacterial endophytes associated with medicinal plants of the Western Ghats, India
Source: FEMS Microbiol Ecol. 2020 Jul 25;96(9):fiaa147. doi: 10.1093/femsec/fiaa147 (PMC7422900; doi:10.1093/femsec/fiaa147)
Supplement: fiaa147_Supplemental_Files [file fiaa147_supplemental_files.zip › Webster_et_al_Culturable_diversity_endophytes_FEMSEC-20-04-0221_Suppl_Tables.docx]

**Supplementary Table 1**. Medicinal plant species collected during this study and their uses in traditional medicine

| **Sample No.** | **Plant species** | **Plant family** | **Endemic** | **IUCN**  **red list** | **Medicinal use** | **Sampling location** |
| --- | --- | --- | --- | --- | --- | --- |
| 1 | *Terminalia paniculate* | Combretaceae | Endemic |  | Diabetes, anemia, cholera, leprosy | **Bisle Ghat region**, Hassan district, Karnataka,  India |
| 2 | *Ventilago* sp. | Rhamnaceae |  |  | Dyspepsia, leprosy, pruritus |  |
| 3 | *Terminalia bellirica* | Combretaceae |  |  | Respiratory tract infections,  Cough and sore throat |  |
| 4 | *Nothapodytes nimmoniana* | Icacinaceae | Endemic |  | Anti-cancer, anti-HIV, antimalarial, antibacterial, antioxidant,  anti-inflammatory, anti-fungal |  |
| 5 | *Garcinia xanthochymus* | Clusiaceae | Endemic |  | Antimicrobial, antidiabetic, antioxidant, nerve growth factor |  |
| 6 | *Aphanamixis polystachya* | Meliaceae | Endemic |  | Astringent, antimicrobial,  treatment of liver/spleen diseases,  rheumatism, tumours |  |
| 7 | *Salacia macrosperma* | Celastraceae | Endemic |  | Gonorrhoea, asthma, itchiness, joint pain (rheumatism), obesity, thirst,  menstrual problems |  |
| 8 | *Garcinia gummi-gutta* | Clusiaceae | Endemic |  | Constipation, oedema, ulcers, haemorrhoids, dysentery, intestinal parasites, irregular menstruation,  open sores, diarrhoea, fever |  |
| 9 | *Memecylon malabaricum* | Melastomataceae | Endemic |  | Skin disorders, stomach disorders,  herpes, chickenpox, leucorrhoea, polyuria, menorrhagia, dysentery, antibacterial |  |
| 10 | *Dysoxylum binectariferum* | Meliaceae | Endemic |  | Skin diseases, inflammation, cardiovascular disorder, neurological disorders, tumours |  |
| 11 | *Kingiodendron pinnatum* | Fabaceae | Endemic | Endangered | Gonorrhoea, catarrhal conditions of Genito-urinary and respiratory tracts |  |
| 12 | *Aristolochia tagala* | Aristolochiaceae | Endemic |  | Gastric stimulant and bitter tonic |  |
| 13 | *Pterocarpus santalinus* | Fabaceae | Endemic | Near threatened | Antihyperglycemic activity, antipyretic, anti-inflammatory, anthelmintic, tonic, haemorrhage, dysentery, aphrodisiac, diaphoretic activities | **Mysore**,  Karnataka, India |
| 14 | *Garcinia indica* | Clusiaceae | Endemic | Vulnerable | Wound healing, prevent chronic disease, reduce allergic reactions,  optimize digestion, protect skin,  boost immune system, relieve pain, eliminate inflammation |  |
| 15 | *Nothapodytes nimmoniana* | Icacinaceae | Endemic |  | Anti-cancer, anti-HIV, antimalarial, antibacterial, antioxidant,  anti-inflammatory, anti-fungal |  |
| 16 | *Coscinium fenestratum* | Menispermaceae | Endemic | Data deficient - thought to be endangered | Anti-microbial, anti-diabetic, anti-inflammatory, antioxidant | **Mangaluru**,  Karnataka, India |
| 17 | *Coix lacryma-jobi* | Poaceae |  |  | Invigorate the spleen function and promote urination, alleviate arthritis, arrest diarrhoea, remove heat and facilitate drainage of pus, antimicrobial |  |
| 18 | *Salacia chinensis* | Celastraceae |  |  | The root extract shows various activities like antioxidant, anticaries, antiulcer, antidiabetic |  |
| 19 | *Calophyllum inophyllum* | Calophyllaceae |  | Least concern | Wounds, ulcers, treat phthisis, orchitis and lung affections, and internally as a purgative, and to treat gonorrhoea |  |
| 20 | *Madhuca insignis* | Sapotaceae | Endemic | Extinct | Antidiabetic, ulcers, hepatic protective, anti-pyretic, analgesic, antioxidant, swelling, inflammation, piles, emetic, dermatological, laxative, tonic, wound healing and headache |  |
| 21 | *Garcinia morella* | Clusiaceae |  |  | Dysentery, gastritis, etc. And is said to have anti inflammatory |  |
| 22 | *Apama siliquosa* | Aristolochiaceae | Endemic |  | Pacifies, remedy for poison bites, stomach pain |  |
| 23 | *Desmodium pulchellum* | Fabaceae |  | Least concern | Cold, fever, malaria, excessive menstrual flow, ulcers, diarrhoea, eye afflictions, bile and liver afflictions. |  |
| 24 | *Barringtonia acutangula* | Lecythidaceae |  | Least concern | Haemolytic disease, abdominal colic, lumbar pain, syphilis, blennorrhoea, febrifuge, malaria, diabetes. |  |
| 25 | *Barringtonia acutangula*  (fruit) | Lecythidaceae |  | Least concern | Wound healing |  |
| 26 | *Alstonia scholaris* | Apocynaceae |  | Least concern | Treat fever, malaria, troubles in digestion, tumours, ulcers and asthma. |  |

**Supplementary Table 2**. Summary of genome assemblies for 26 medicinal plant endophytes

| **ME genome** | **Species** | **Genome size (Mbp)** | **Sequencing depth (X)** | **Contig Number**  **(>1000 bp)** | **N_50_ (bp)** | **G + C content (%)** |
| --- | --- | --- | --- | --- | --- | --- |
| ME5 | *Bacillus* sp. | 5.49 | 51 | 116 | 97,952 | 35.20 |
| ME7 | *Aureimonas* sp. | 4.38 | 55 | 45 | 301,236 | 66.89 |
| ME12 | *Curtobacterium* sp. | 3.71 | 55 | 74 | 85,768 | 70.91 |
| ME13 | *Enterobacter bugandensis* | 4.65 | 54 | 62 | 132,712 | 56.15 |
| ME25 | *Bacillus taxi* | 6.16 | 41 | 236 | 55,441 | 37.50 |
| ME26 | *Curtobacterium* sp. | 3.57 | 52 | 103 | 70,481 | 71.98 |
| ME27 | *Acinetobacter lactucae* | 3.89 | 70 | 27 | 328,038 | 38.76 |
| ME30 | *Klebsiella pneumoniae* | 5.52 | 54 | 100 | 125,281 | 56.95 |
| ME34 | *Enterobacter bugandensis* | 4.65 | 55 | 69 | 111,148 | 56.14 |
| ME35 | *Bacillus* *licheniformis* | 4.25 | 52 | 43 | 238,213 | 46.05 |
| ME39 | *Bacillus* *aryabhattai* | 5.63 | 62 | 50 | 571,092 | 37.52 |
| ME40 | *Bacillus* sp. | 5.16 | 51 | 37 | 382,796 | 38.00 |
| ME42 | *Bacillus* *aryabhattai* | 5.48 | 58 | 49 | 408,314 | 37.61 |
| ME43 | *Serratia* sp. | 5.43 | 55 | 514 | 16,042 | 59.36 |
| ME44 | *Enterobacter asburiae* | 4.52 | 57 | 60 | 124,981 | 56.05 |
| ME47 | *Serratia* sp. | 5.28 | 66 | 63 | 156,947 | 59.37 |
| ME55 | *Stenotrophomonas pavanii* | 4.39 | 79 | 61 | 121,849 | 67.32 |
| ME63 | *Pseudomonas* sp. | 6.01 | 58 | 710 | 11,254 | 63.40 |
| ME73 | *Klebsiella variicola* | 5.52 | 57 | 73 | 178,119 | 57.40 |
| ME75 | *Bacillus sp.* | 5.73 | 45 | 143 | 163,647 | 37.71 |
| ME76 | *Bacillus sp.* | 5.74 | 49 | 205 | 92,118 | 37.70 |
| ME78 | *Bacillus sp.* | 5.63 | 54 | 82 | 154,552 | 35.03 |
| ME79 | *Klebsiella variicola* | 5.49 | 54 | 203 | 44,167 | 57.40 |
| ME81 | *Pantoea* sp. | 5.55 | 45 | 364 | 28,575 | 53.81 |
| ME86 | *Klebsiella variicola* | 7.23 | 38 | 734 | 35,941 | 56.18 |
| ME94 | *Methylobacterium radiotolerans* | 6.41 | 35 | 114 | 149,973 | 71.47 |

ME = medicinal plant endophyte
