# Supplementary figures and images for: Culturable diversity of bacterial endophytes associated with medicinal plants of the Western Ghats, India
Source: FEMS Microbiol Ecol. 2020 Jul 25;96(9):fiaa147. doi: 10.1093/femsec/fiaa147 (PMC7422900; doi:10.1093/femsec/fiaa147)

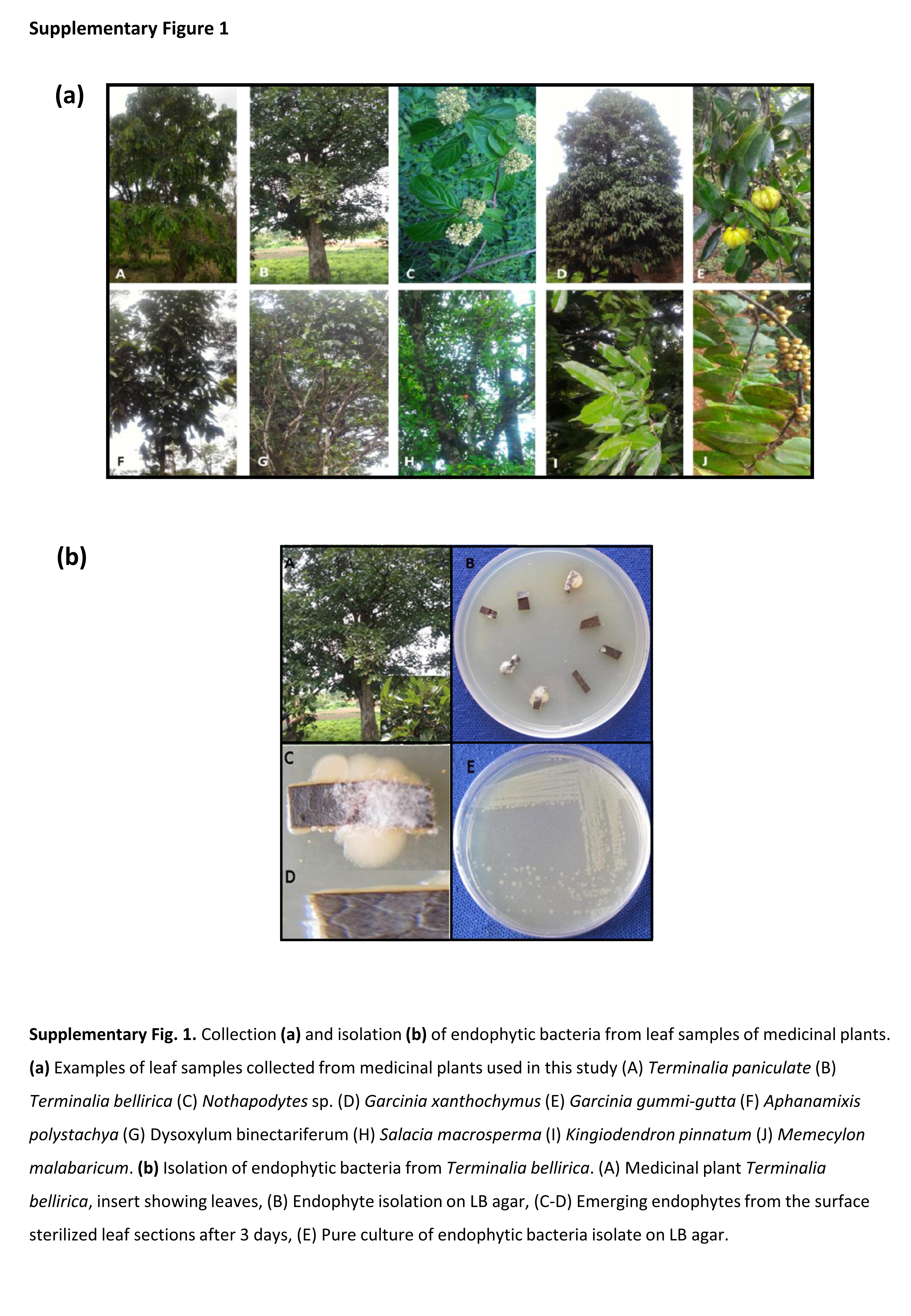

Supplement: fiaa147_Supplemental_Files [file fiaa147_supplemental_files.zip › S1.TIF]

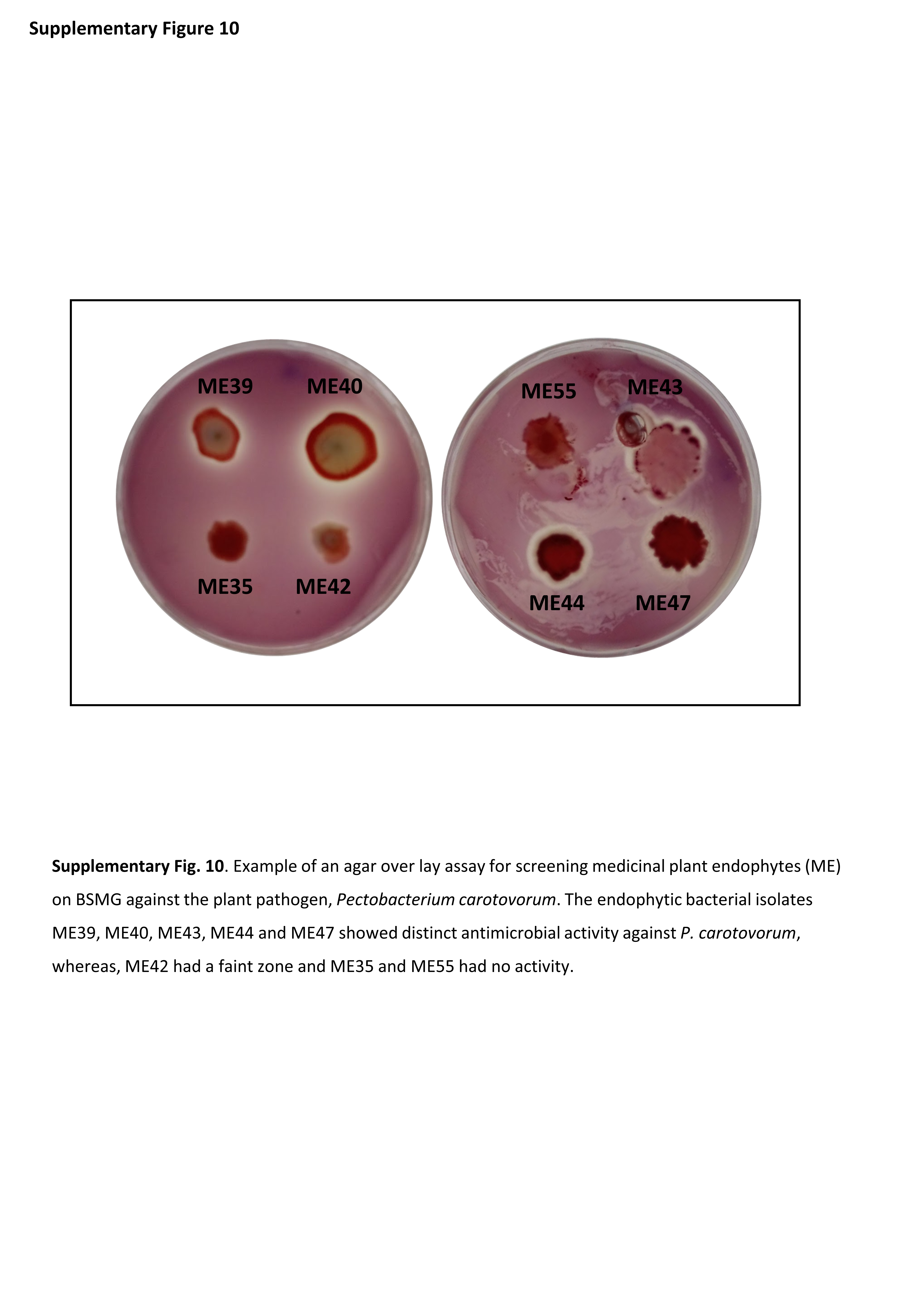

Supplement: fiaa147_Supplemental_Files [file fiaa147_supplemental_files.zip › S10.TIF]

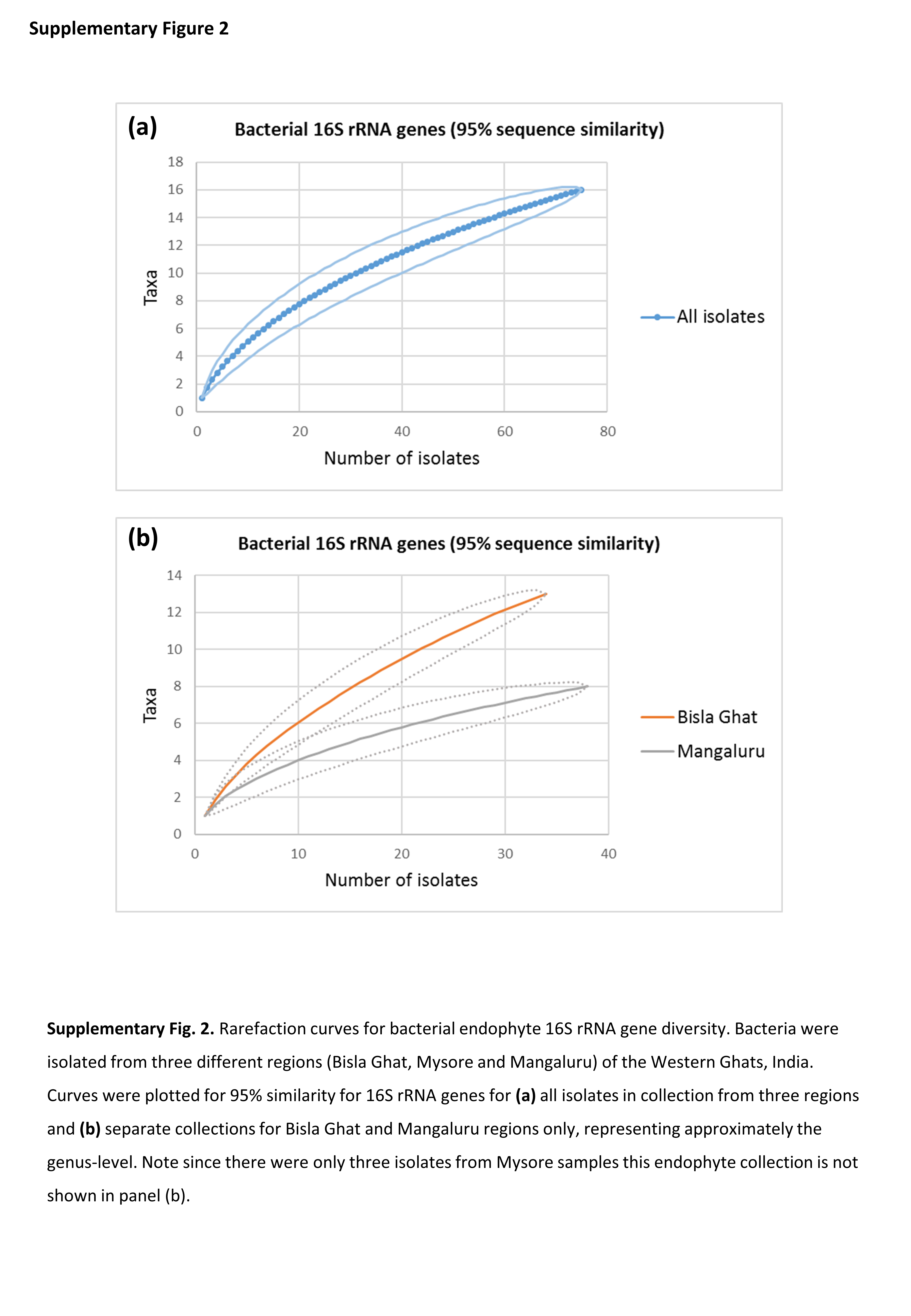

Supplement: fiaa147_Supplemental_Files [file fiaa147_supplemental_files.zip › S2.TIF]

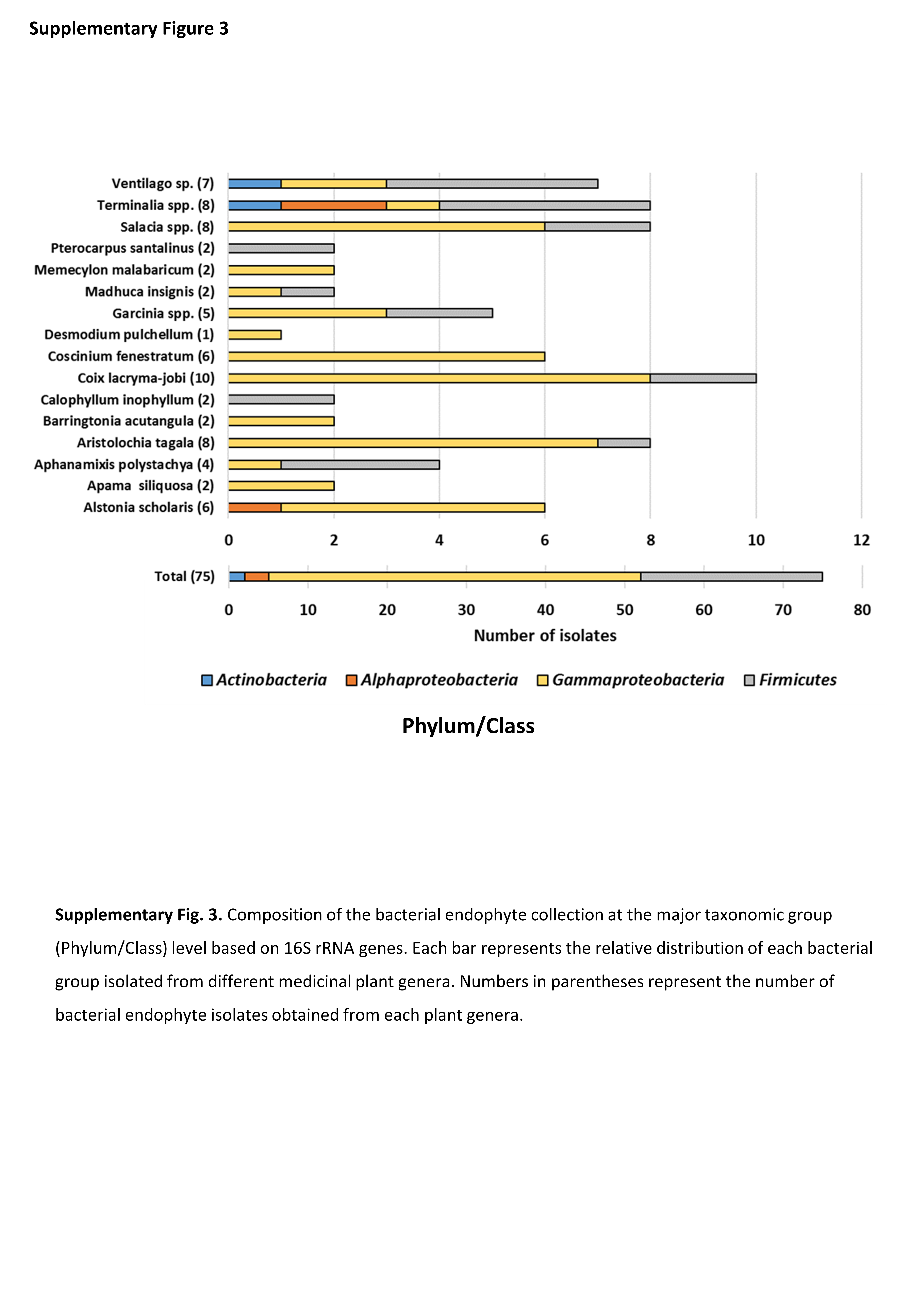

Supplement: fiaa147_Supplemental_Files [file fiaa147_supplemental_files.zip › S3.TIF]

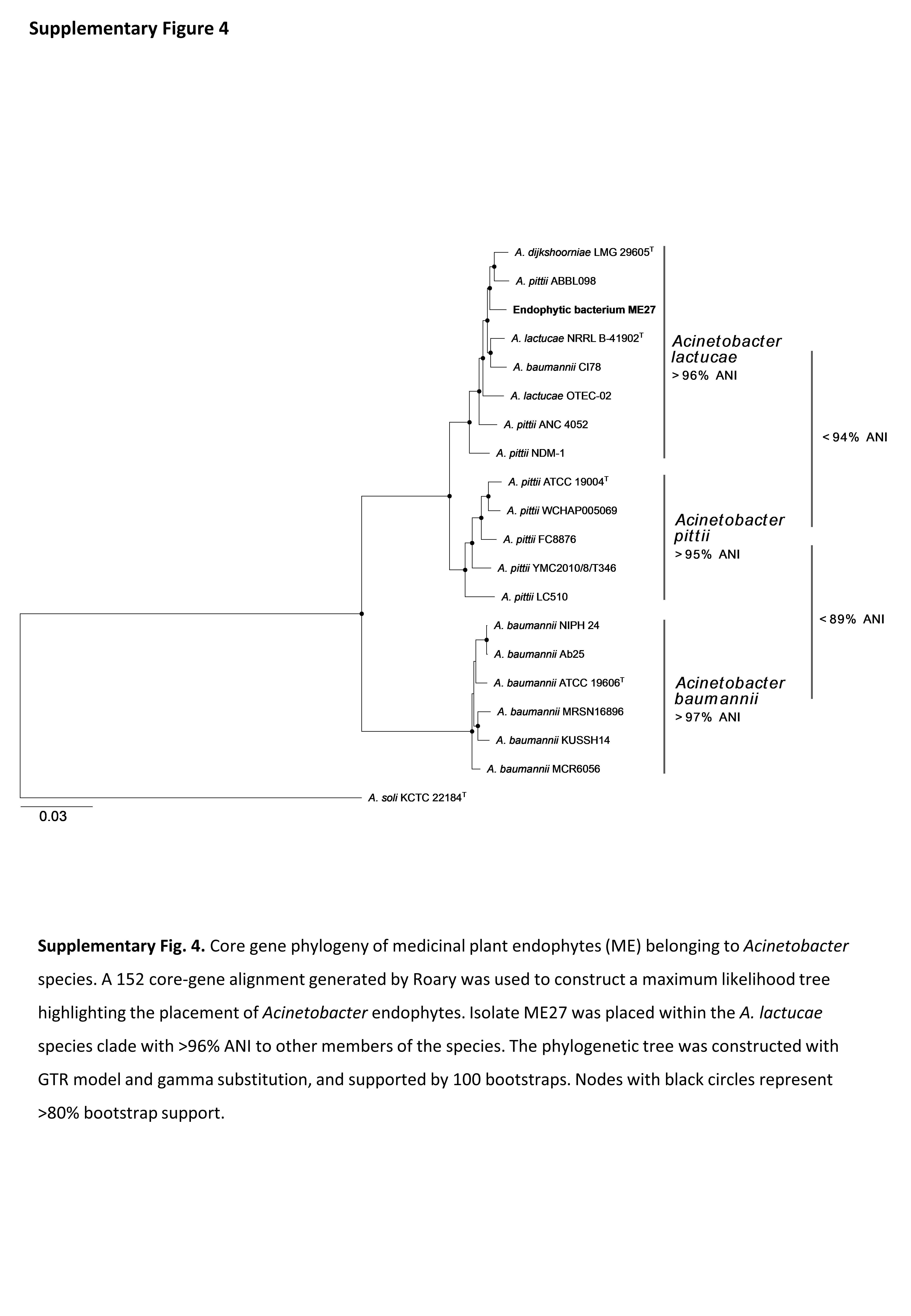

Supplement: fiaa147_Supplemental_Files [file fiaa147_supplemental_files.zip › S4.TIF]

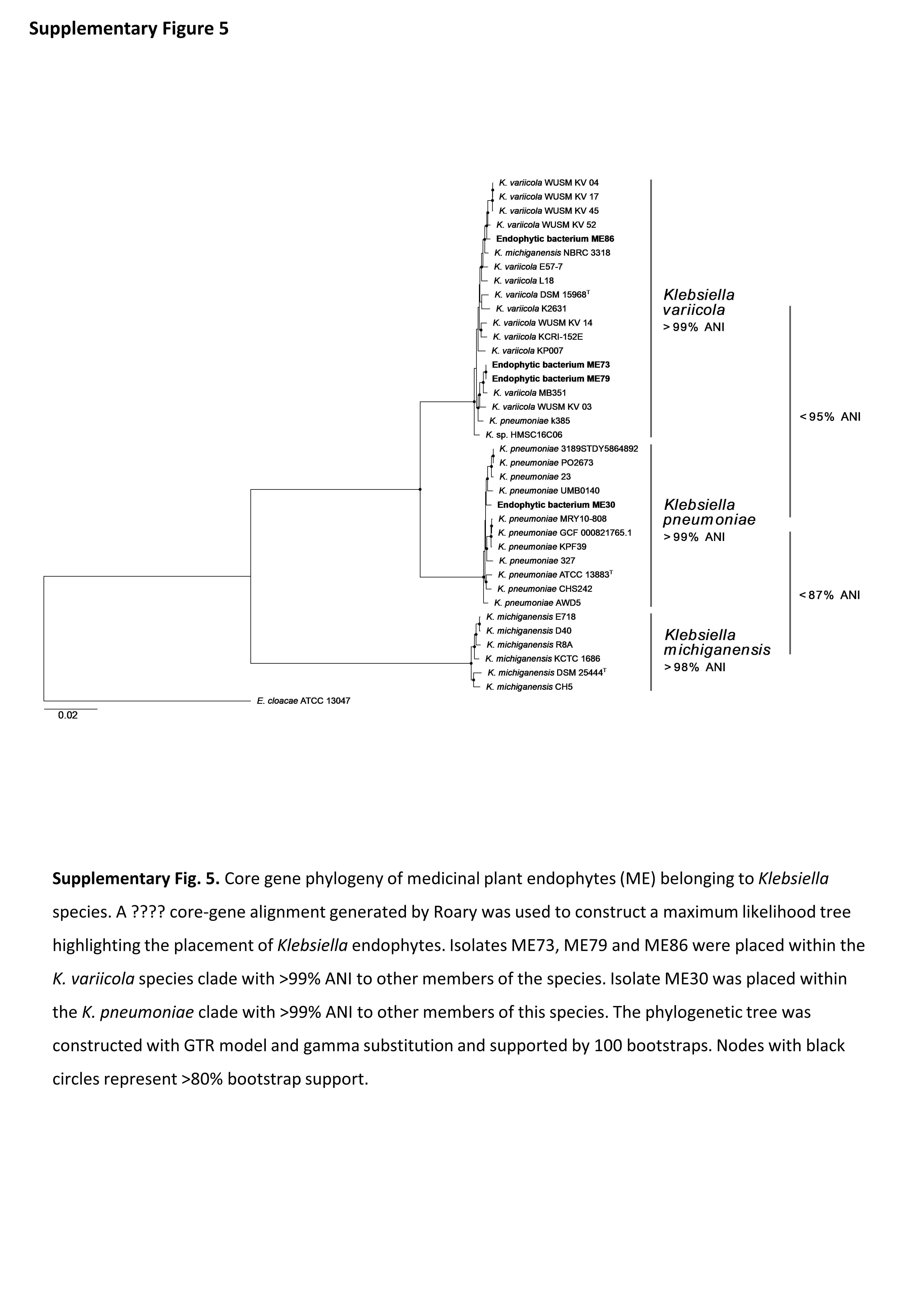

Supplement: fiaa147_Supplemental_Files [file fiaa147_supplemental_files.zip › S5.TIF]

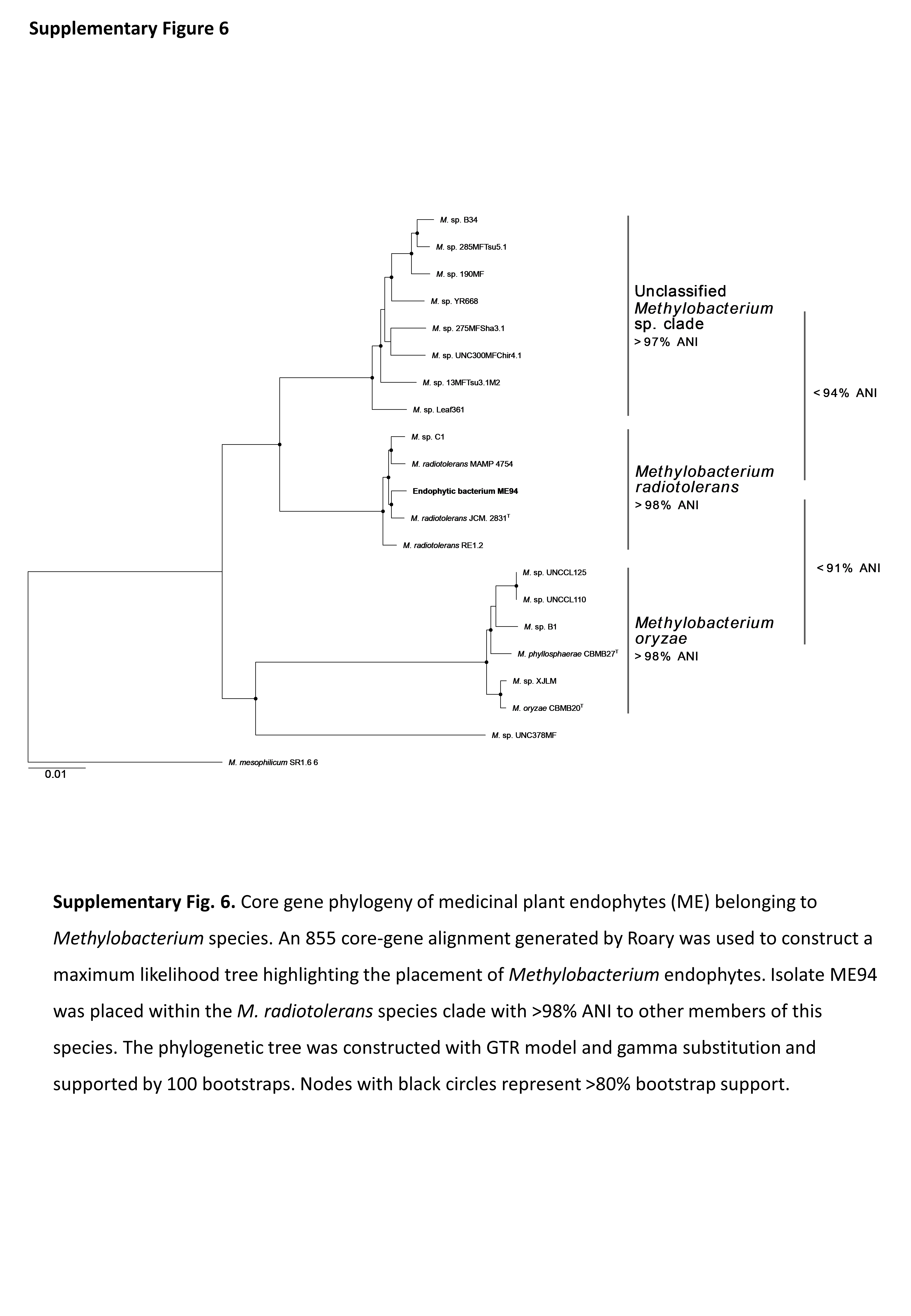

Supplement: fiaa147_Supplemental_Files [file fiaa147_supplemental_files.zip › S6.TIF]

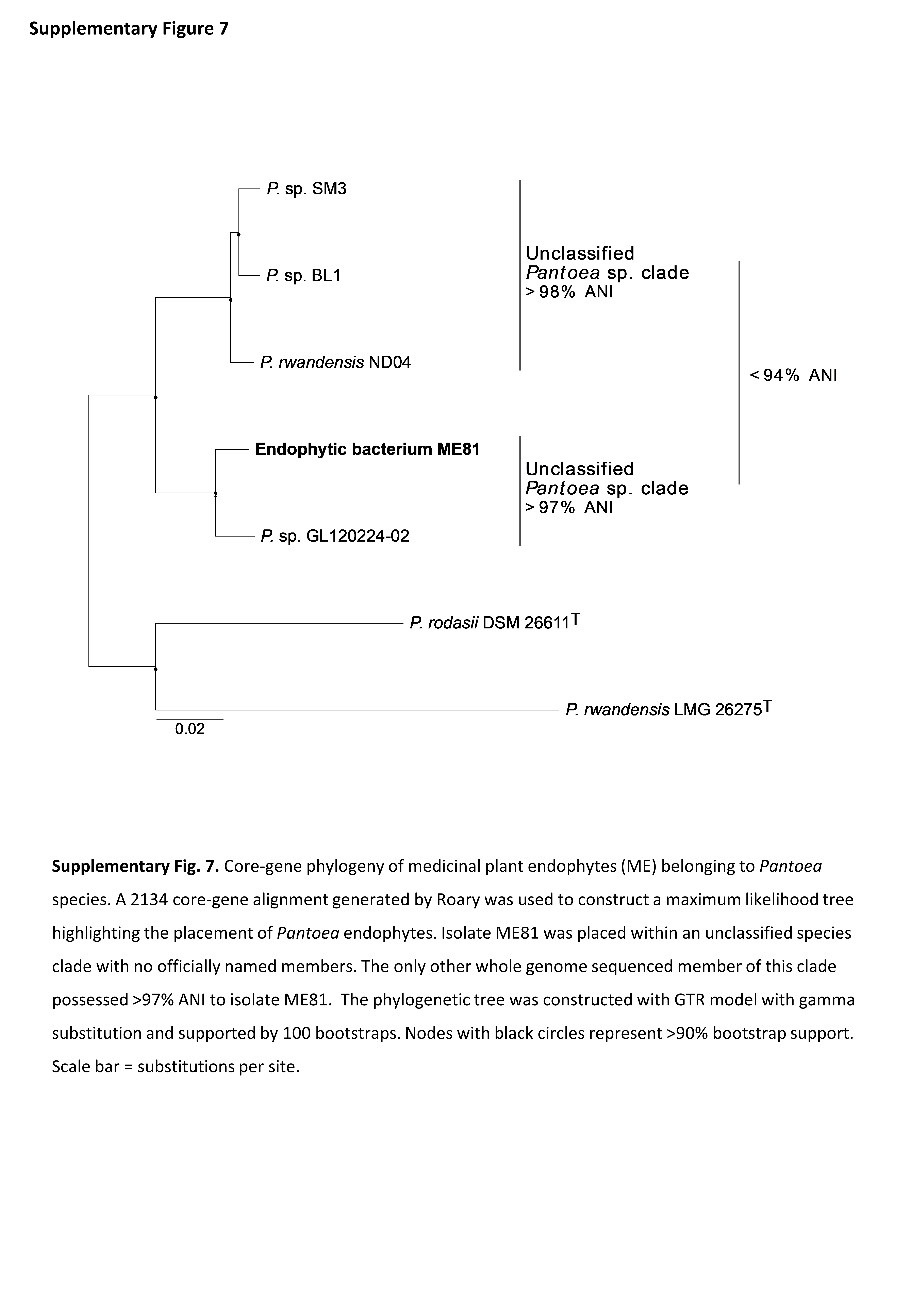

Supplement: fiaa147_Supplemental_Files [file fiaa147_supplemental_files.zip › S7.TIF]

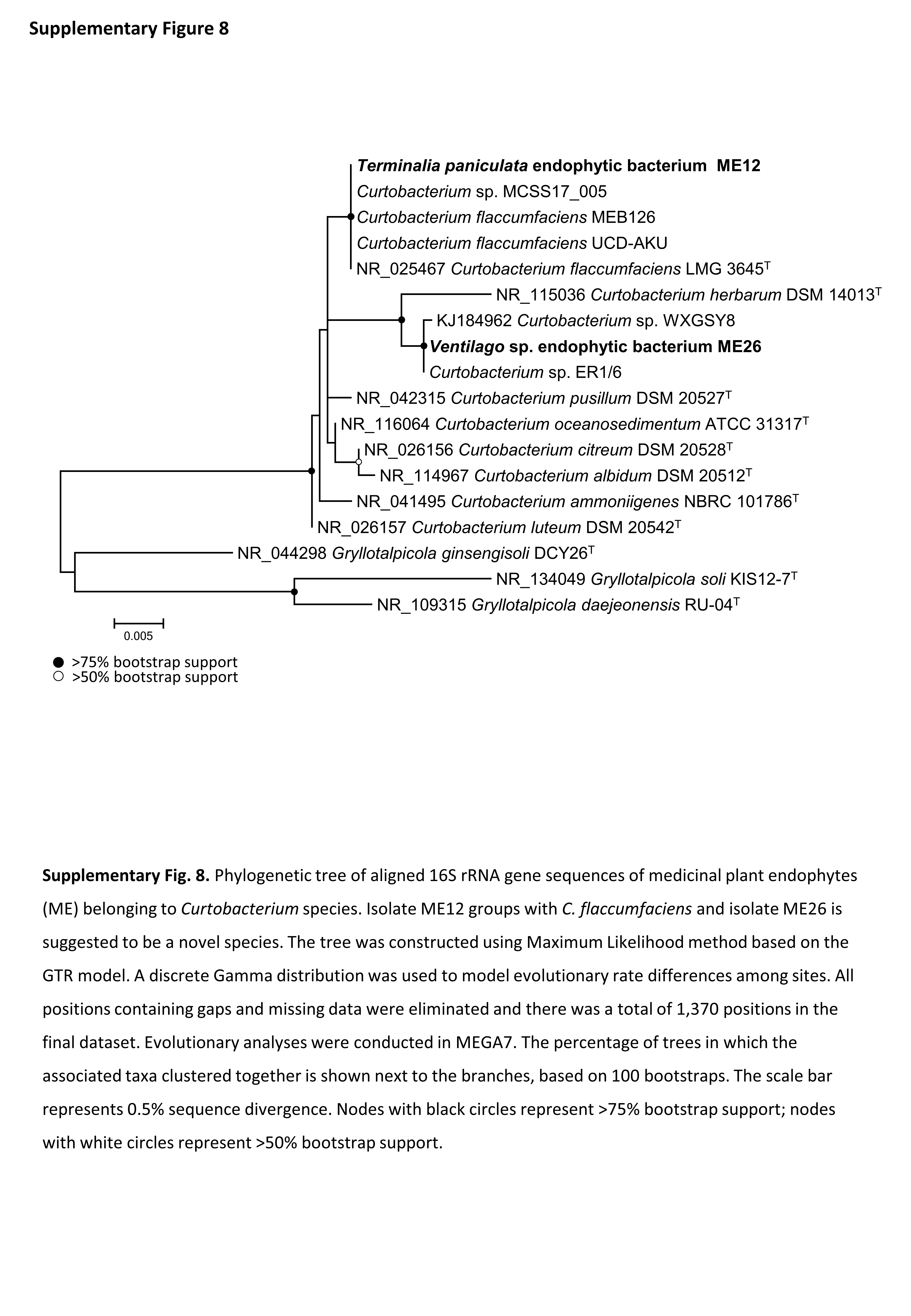

Supplement: fiaa147_Supplemental_Files [file fiaa147_supplemental_files.zip › S8.TIF]

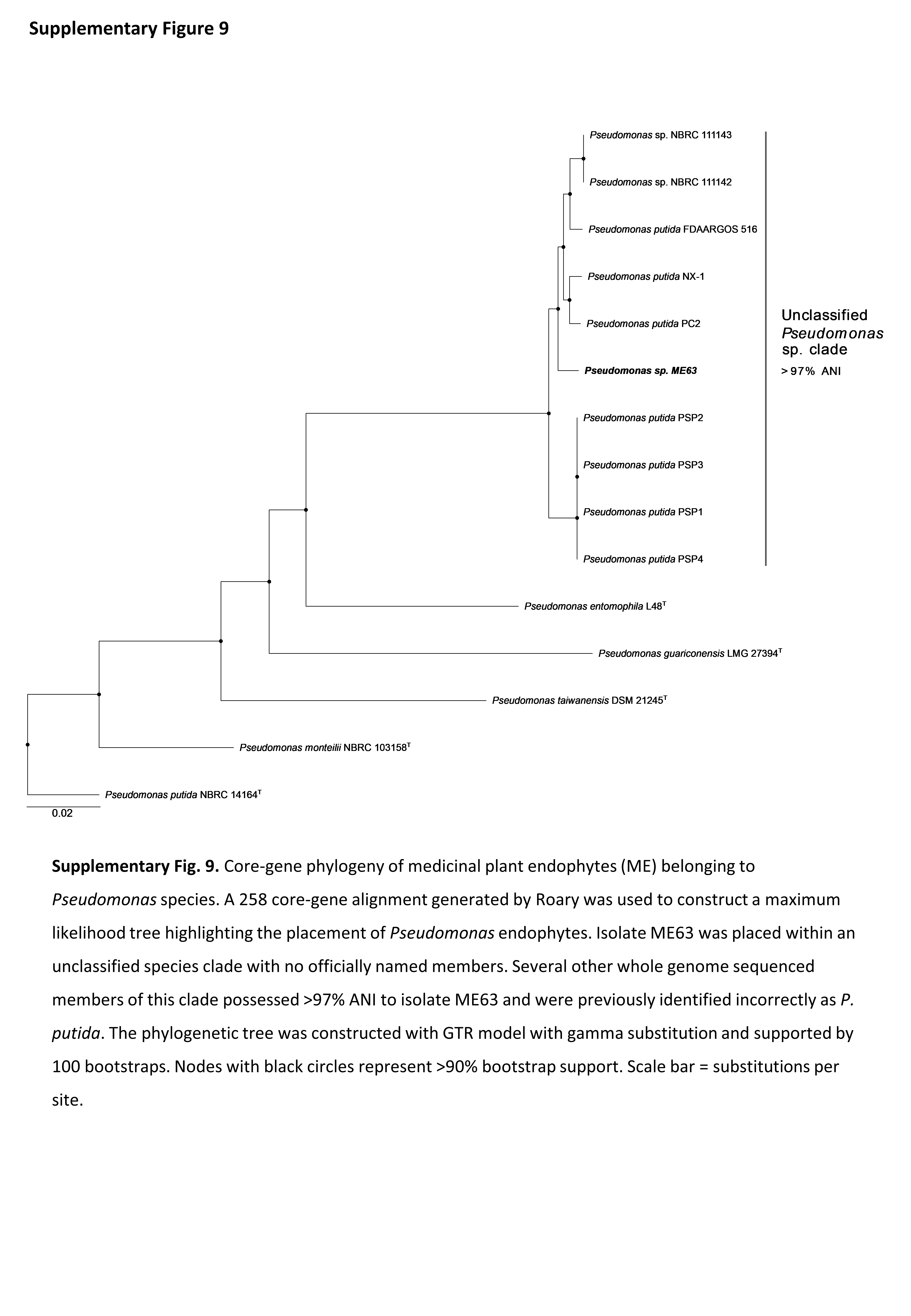

Supplement: fiaa147_Supplemental_Files [file fiaa147_supplemental_files.zip › S9.TIF]
